# Supplementary material for: Stress signaling in breast cancer cells induces matrix components that promote chemoresistant metastasis
Source: EMBO Mol Med. 2018 Sep 6;10(10):e9003. doi: 10.15252/emmm.201809003 (PMC6180299; doi:10.15252/emmm.201809003)
Supplement: Supplementary file 1 — Appendix [file EMMM-10-e9003-s001.pdf]

Appendix

Stress signaling in breast cancer cells induces matrix components that promote chemoresistant metastasis

Jacob Insua-Rodríguez, Maren Pein, Tsunaki Hongu, Jasmin Meier, Arnaud Descot, Camille M. Lowy, Etienne De Braekeleer, Hans-Peter Sinn, Saskia Spaich, Marc Sütterlin, Andreas Schneeweiss and Thordur Oskarsson

Table of Content

Appendix Figure S1. JNK signaling in samples from breast cancer patients

Appendix Figure S2. Generation of highly metastatic SUM159-LM1 breast cancer cells.

Appendix Figure S3. JNK signaling in xenograft models of breast cancer metastasis

Appendix Figure S4. Association between JNK signaling and gene expression signatures

Appendix Figure S5. GSEA of basal cell genes in oncospheres

Appendix Figure S6. Role of JNK signaling in tumor initiation

Appendix Figure S7. *SPP1* and *TNC* knockdowns used in metastasis assays

Appendix Figure S8. *SPP1* expression in chemotherapy-treated breast cancer cells

Appendix Figure S9. Experimental setup to address chemotherapy response of *SPP1* deficient mammary tumors and metastases in mice

Appendix Figure S10. TNC knockdown in breast cancer cells

Appendix Figure S11. Apoptosis in mammary tumors treated with combination of JNKi and PAX

Appendix Figure S12. Association between JNK activity and diverse therapy resistance signatures

Appendix Table S1. Clinical features of human breast tumor samples

Appendix Table S2. Association analysis between JNK activity in TMA samples and clinical parameters

Appendix Table S3. *P* values for all figures.

Appendix Supplementary Methods

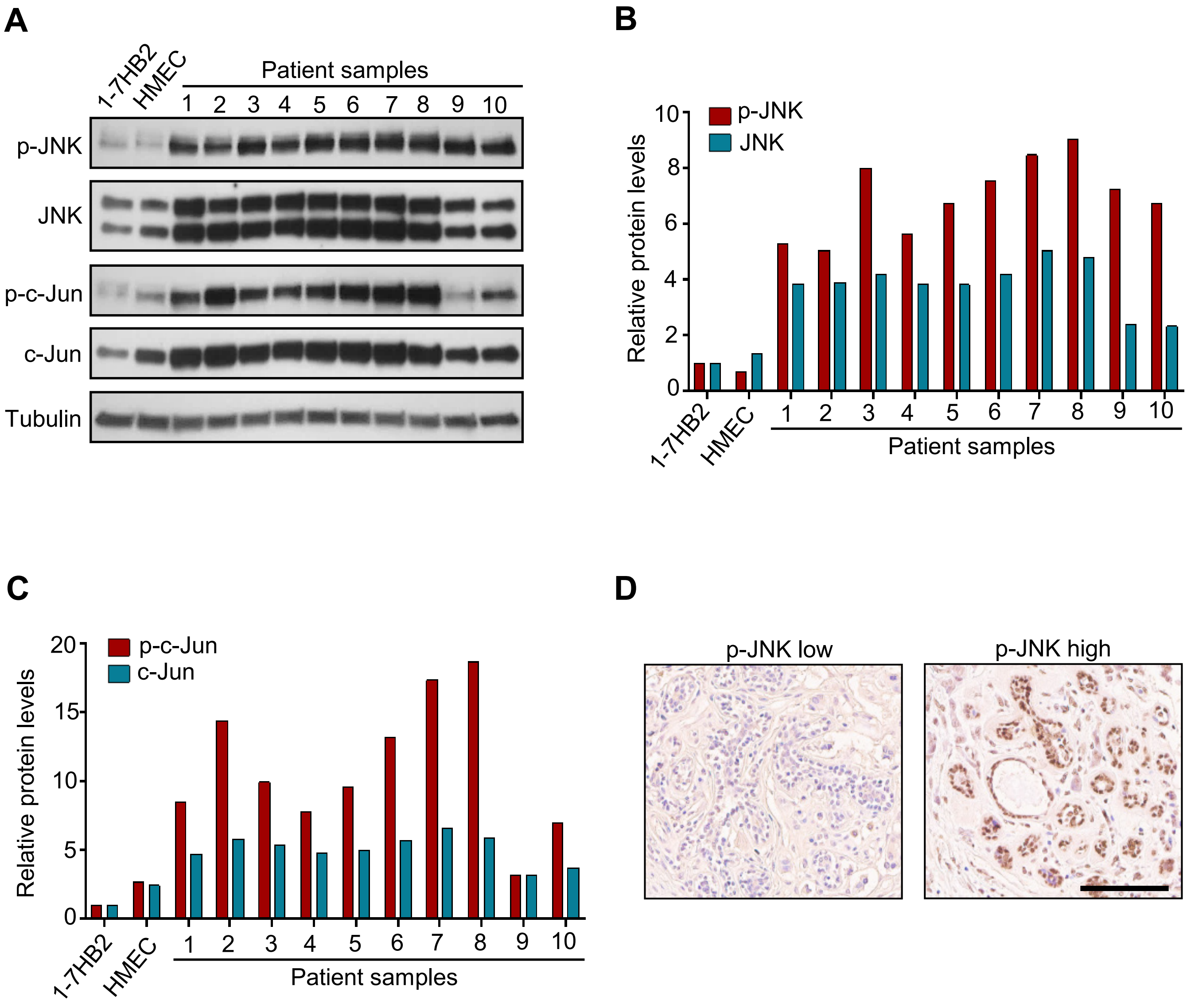

Appendix Figure S1. JNK signaling in samples from breast cancer patients. (**A**) Western blot analysis of phosphorylated JNK (p-JNK, Thr183/Tyr185), JNK, phosphorylated c-Jun (p-c-Jun, Ser63) and c-Jun in normal human mammary epithelial cells (1-7HB2, HMEC) and in breast cancer cells isolated from patient samples. Samples 1-4 are derived from pleural effusions and samples 5-10 are derived from ascites from breast cancer patients with metastasis. Tubulin is shown as a loading control. (**B** and **C**) Quantification of p-JNK, JNK (B) and p-c-Jun, c-Jun (C) in Western blot of panel A. Shown are protein levels normalized to tubulin and relative to protein levels in 1-7HB2 cells. (**D**) Representative images from tissue microarray showing low and high p-JNK expression in breast cancer. Scale bar,100 µm.

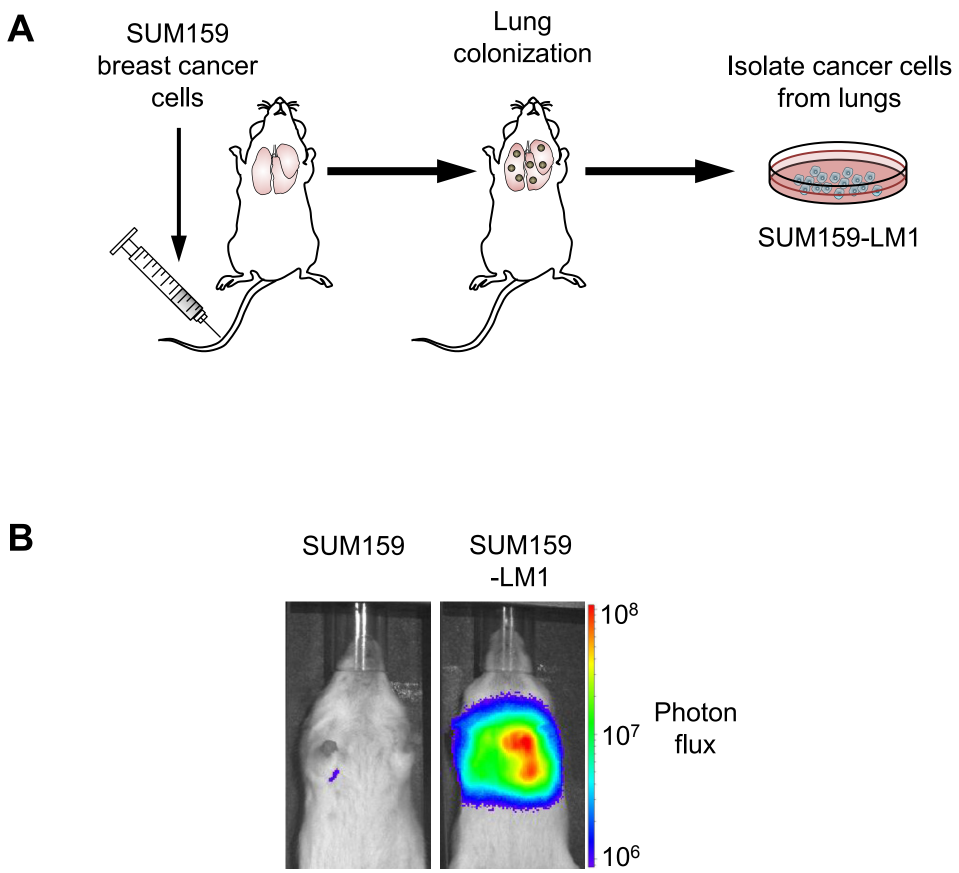

Appendix Figure S2. Generation of highly metastatic SUM159-LM1 breast cancer cells. (**A**) Schematic diagram of strategy used to generate SUM159-LM1, a lung metastatic derivative of SUM159 breast cancer cells. (**B**) Representative bioluminescence of mice injected intravenously with indicated breast cancer cells. Mice were imaged four weeks post injection.

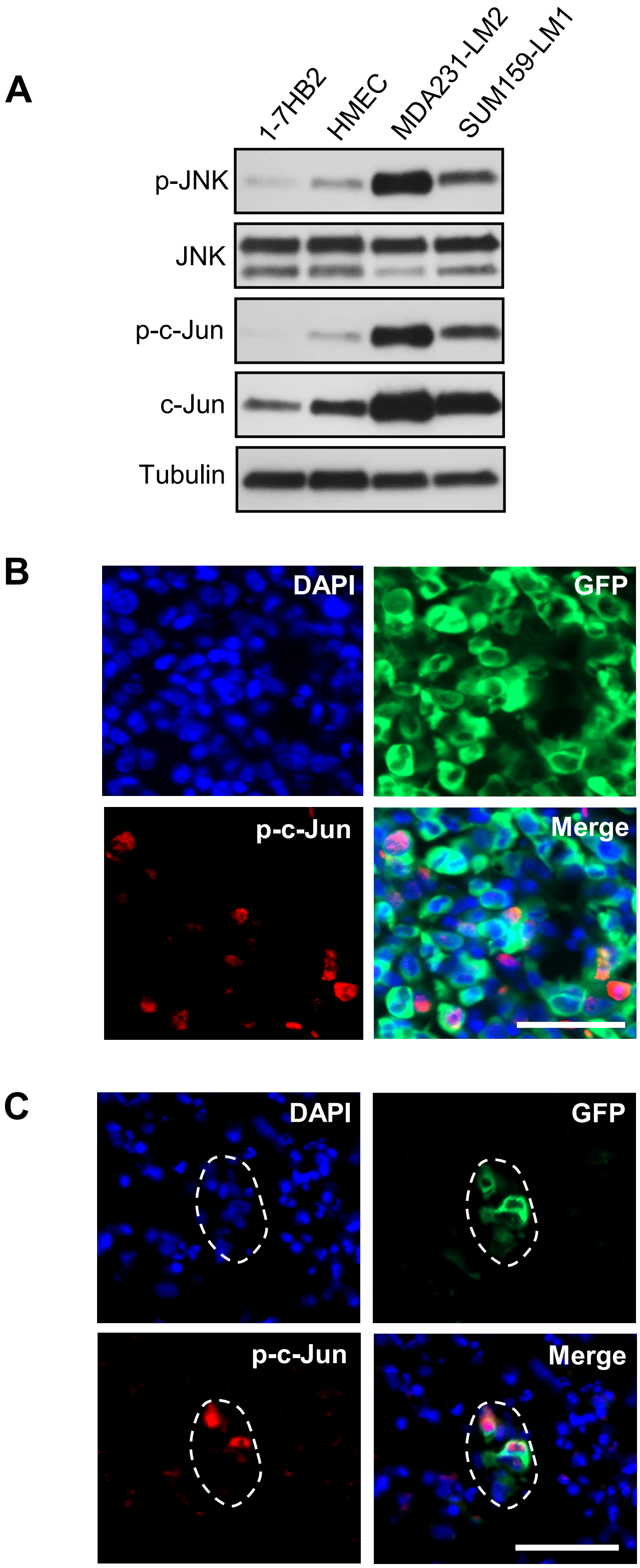

Appendix Figure S3. JNK signaling in xenograft models of breast cancer metastasis. (**A**) Western blot analysis of p-JNK, JNK, p-c-Jun and c-Jun in MDA231-LM2 and SUM159-LM1 breast cancer cell lines compared to normal mammary epithelial cells (1-7HB2, HMEC). Loading control, tubulin. (**B** and **C**) Immunofluorescence analysis detecting p-c-Jun positive cancer cells in a matched mammary tumor (B) and micrometastasis (C). Images are representative examples from an NSG mouse injected with MDA231-LM2 cells to the mammary fat pad. Scale bars, 50 µm.

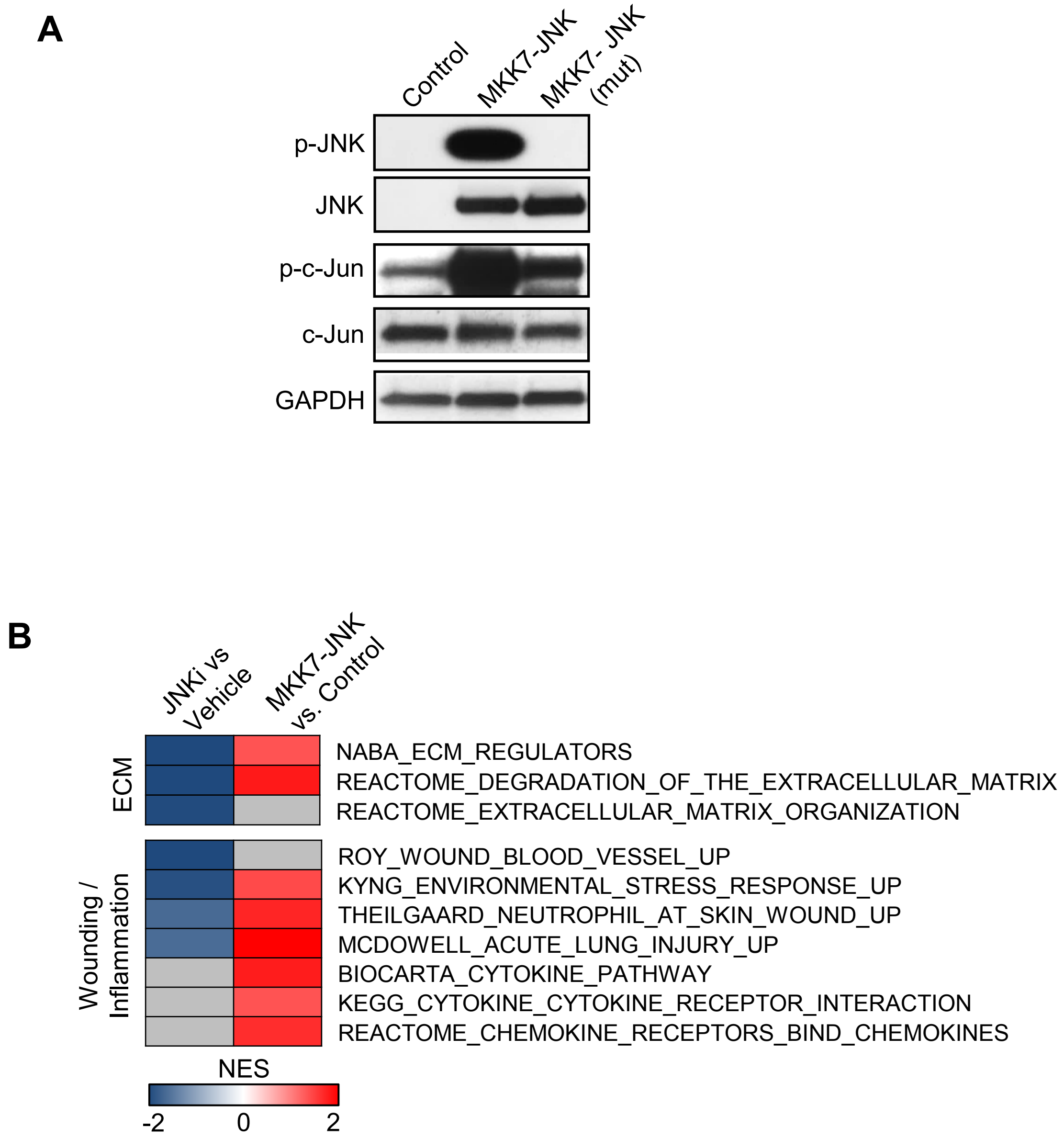

Appendix Figure S4. Association between JNK signaling and gene expression signatures. (**A**) Western blot analysis of JNK and c-Jun proteins and phosphorylated JNK (p-JNK, Thr183/Tyr185) and c-Jun (p-c-Jun, Ser63) in MDA231-LM2 breast cancer cells expressing constitutively active JNK (MKK7-JNK) or mutated inactive JNK (MKK7-JNK(mut)). The blot shows ectopic JNK and p-JNK. GAPDH was used as a loading control. (**B**) Heatmap representing normalized enrichment scores (NES) of ECM, wound healing and inflammation signatures (Naba et al, 2012, McDowell et al, 2003, Theilgaard-Monch et al, 2004, Kyng et al, 2005, Roy et al, 2007) retrieved from the C2 collection of the GSEA Molecular Signatures Database (MSigDB) in MDA231-LM2 cells treated with JNK inhibitor (JNKi) or overexpressing active JNK. BH-*P*-values < 0.05 and FDR < 0.1 was considered statistically significant. Gray boxes depict not significantly changed score.

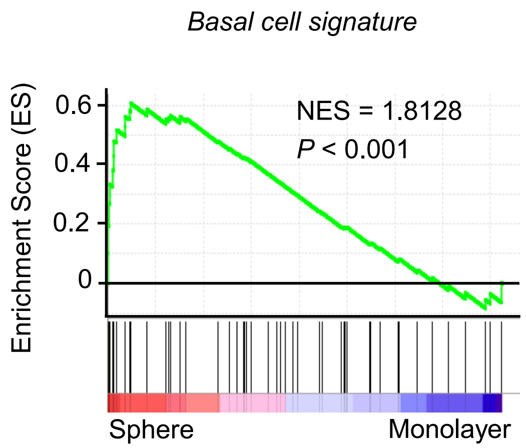

Appendix Figure S5. GSEA of basal cell genes in oncospheres. Enrichment of basal cell signature (Huper & Marks, 2007) in MDA231-LM2-derived oncospheres. NES, normalized enrichment score. *P* value was determined by random-permutation test.

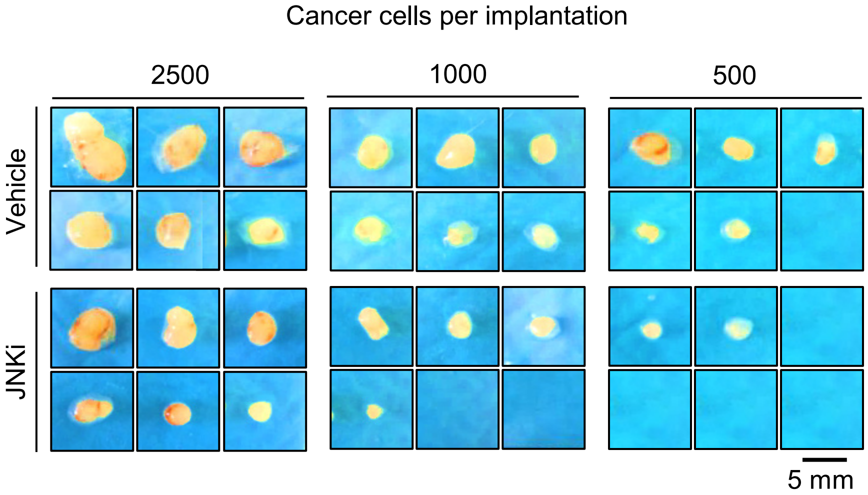

Appendix Figure S6. Role of JNK signaling in tumor initiation. Representative images of tumor initiation from NSG mice injected subcutaneously with MDA231-LM2 breast cancer cells and treated with JNK inhibitor (JNKi) (*n* = 3 mice per group, two implantations per mouse).

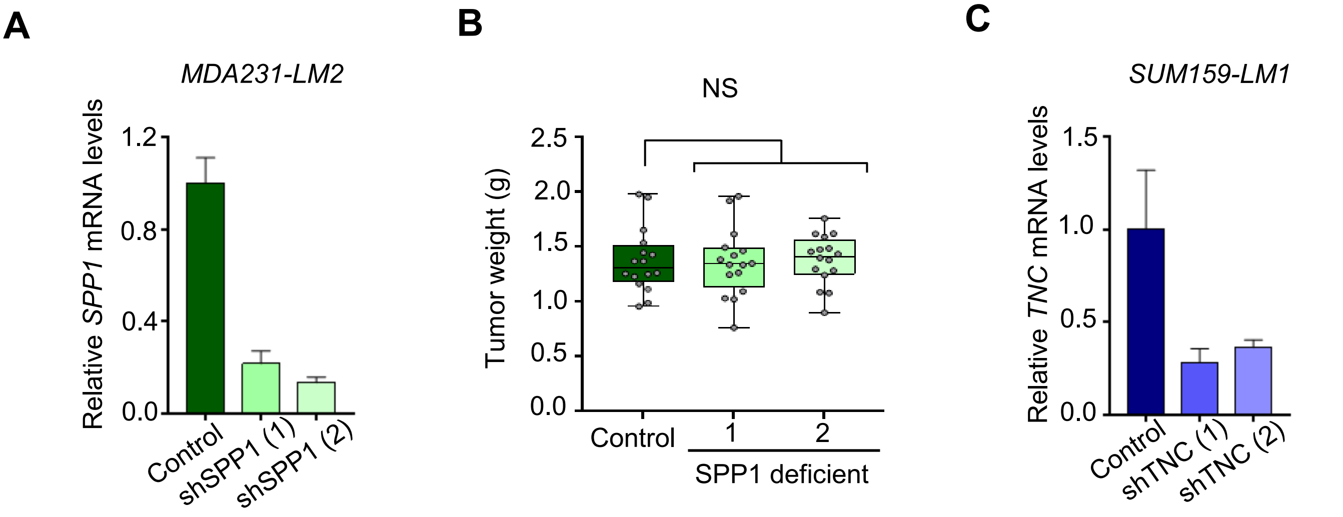

Appendix Figure S7. *SPP1* and *TNC* knockdowns used in metastasis assays. (**A**) *SPP1* expression in control and shSPP1-transduced MDA231-LM2 breast cancer cells. Two independent hairpins against SPP1 were used. (**B**) Mammary tumor weight in *Spp1*$^{+/-}$ mice implanted with MDA231-LM2 cells expressing control shRNA (control), and *Spp1*$^{-/-}$ mice implanted with MDA231-LM2 cells independently expressing one of two different SPP1 shRNAs (SPP1-deficient). Boxes show the median with upper and lower quartiles. Whiskers represent minimum and maximum values. *N* = 14-16 mice per group. *P* values were determined by a two-tailed Mann-Whitney test. NS, not statistically significant. (**C**) Expression of TNC in SUM159-LM1 breast cancer cells transduced with control or TNC-targeting shRNA. Two independent hairpins were used against TNC. Values in panels A and C are means from triplicates +/- SD.

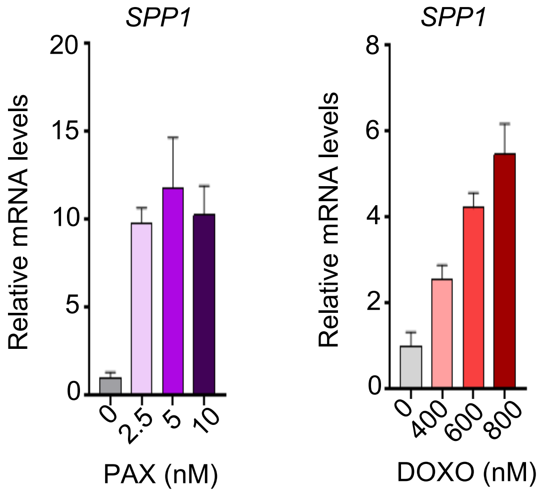

Appendix Figure S8. *SPP1* expression in chemotherapy-treated breast cancer cells. *SPP1* expression in MDA231-LM2 cells after treatment with incremental dosage of paclitaxel (PAX) and doxorubicin (DOXO). Values are means from triplicates +/- SD.

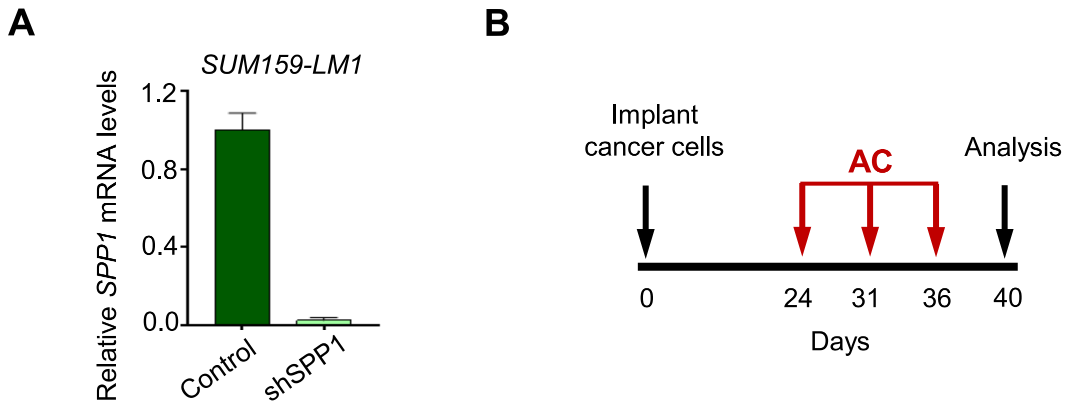

Appendix Figure S9. Experimental setup to address chemotherapy response of *SPP1* deficient mammary tumors and metastases in mice. (**A**) Expression of *SPP1* in control and shSPP1-transduced SUM159-LM1 cells. Values are mean from triplicate qPCR experiments +/- SD. (**B**) Treatment schedule of mice after cancer cell implantation in the mammary gland to address the role of SPP1 in resistance to the combination of doxorubicin (Adriamycin) and cyclophosphamide (AC regimen).

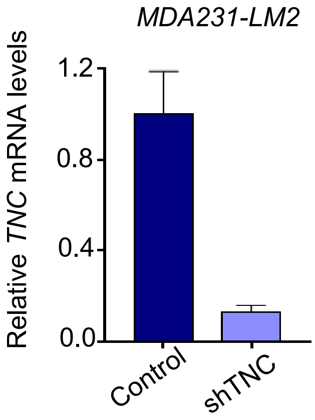

Appendix Figure S10. TNC knockdown in breast cancer cells. TNC expression in MDA231-LM2 cancer cells transduced with control and TNC-targeting shRNA. Expression was determined by qPCR and is shown as mean of triplicates +/- SD.

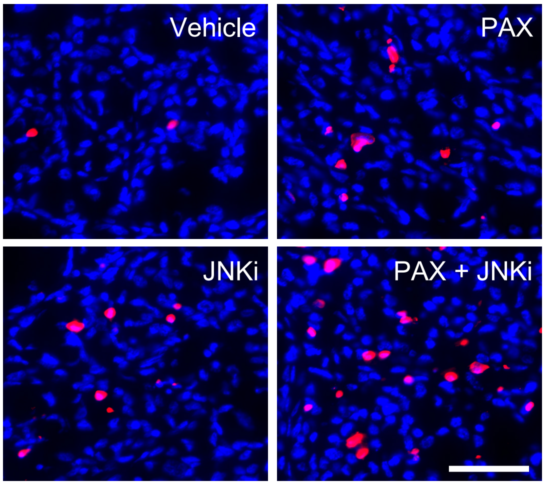

Appendix Figure S11. Apoptosis in mammary tumors treated with combination of JNKi and PAX. Representative images from TUNEL analysis in MDA231-LM2 mammary tumors treated with the indicated therapies. TUNEL-stained cells, red, DAPI-stained nuclei, blue. Scale bar, 50 μm.

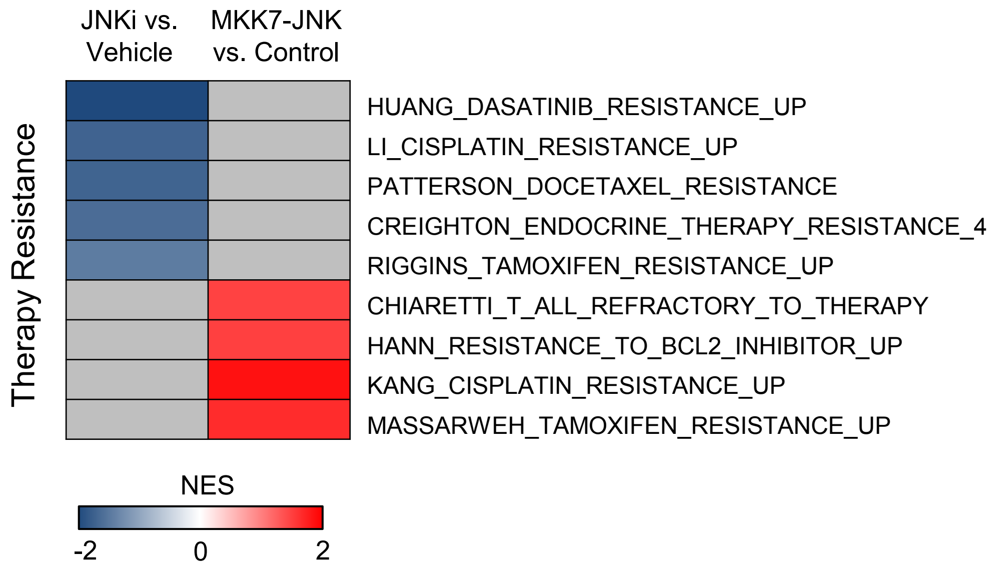

Appendix Figure S12. Association between JNK activity and diverse therapy resistance signatures. Heatmap representing normalized enrichment scores (NES) of therapy resistance signatures from different studies (Huang et al, 2007, Li et al, 2007, Patterson et al, 2006, Creighton et al, 2008, Riggins et al, 2008, Kang et al, 2004, Massarweh et al, 2008, Hann et al, 2008, Chiaretti et al, 2004) retrieved from the C2 collection of the GSEA Molecular Signatures Database (MSigDB) in MDA231-LM2 breast cancer cells treated with JNKi or expressing active JNK. BH-*P*-values < 0.05 and FDR < 0.1 was considered statistically significant. Gray bars, not statistically significant.

Appendix Table S1. Clinical features of human breast tumor samples. Annotated data on tumor samples that were classified according to p-JNK status for Kaplan-Meier analysis (Figure 1A). F, female; IDC, invasive ductal carcinoma; ILC, invasive lobular carcinoma; ER, estrogen receptor status; PR, progesterone receptor status; HER2, human epidermal growth factor receptor 2 (HER2) status; FISH, fluorescence in-situ hybridization; p-JNK, phosphorylated JNK (Thr183/Tyr185); NA, not available; TNM, staging system of malignant tumors (T, tumor size; N, lymph node status, M, distant metastasis). Survival values refer to time after diagnosis.

Patient	Sex	Age	Sample type	Pathology	Grade	Stage	TNM	ER	PR	HER2	HER2 (FISH)	Patient status	Survival (months)	p-JNK status
1	F	82	Breast tumor	IDC	II	2A	T2N0M0	-	-	+	-	Deceased	54	Low
2	F	47	Breast tumor	IDC	I-II	3A	T3N2M0	+	+	-	-	Deceased	32	High
3	F	31	Breast tumor	IDC with ILC	I-II	2A	T2N0M0	+	+	-	-	Deceased	147	Low
4	F	37	Breast tumor	IDC	II-III	2A	T2N0M0	+	+	-	-	Deceased	93	High
5	F	51	Breast tumor	IDC	II	3A	T1N2M0	-	-	-	-	Deceased	18	High
6	F	48	Breast tumor	IDC	II	3C	T2N3M0	-	-	-	-	Deceased	11	High
7	F	68	Breast tumor	IDC	I	2A	T1N1M0	-	-	-	-	Deceased	82	Low
8	F	73	Breast tumor	IDC with ILC	II-III	3A	T2N2M0	+	+	-	-	Deceased	114	Low
9	F	54	Breast tumor	IDC	II	2A	T2N0M0	-	-	-	-	Deceased	97	Low
10	F	33	Breast tumor	IDC	II	3A	T2N2M0	NA	NA	NA	NA	Deceased	19	High
11	F	40	Breast tumor	IDC	I	2B	T2N1M0	-	-	+	+	Deceased	23	High
12	F	46	Breast tumor	IDC	II	3A	T2N2M0	+	-	+	+	Deceased	7	Low
13	F	48	Breast tumor	IDC	II	3A	T2N2M0	NA	+	-	-	Deceased	125	Low
14	F	45	Breast tumor	IDC	I-II	2A	T1N1M0	-	-	-	+	Deceased	47	Low
15	F	75	Breast tumor	IDC	II-III	3A	T2N2M0	+	+	-	-	Deceased	53	Low
16	F	76	Breast tumor	IDC	II	2A	T2N0M0	-	-	+	+	Deceased	77	Low
17	F	78	Breast tumor	IDC	II	2A	T2N0M0	+	-	-	-	Deceased	92	Low
18	F	55	Breast tumor	IDC	I	2B	T2N1M0	+	+	-	-	Deceased	31	Low
19	F	50	Breast tumor	IDC	II	3C	T2N3M0	NA	NA	NA	NA	Deceased	78	Low
20	F	53	Breast tumor	IDC	II	3A	T2N2M0	+	+	+	-	Deceased	63	Low
21	F	63	Breast tumor	IDC	II	2A	T1N1M0	-	-	-	-	Deceased	35	Low
22	F	40	Breast tumor	IDC	I-II	2A	T2N0M0	+	+	-	-	Deceased	44	High
23	F	74	Breast tumor	IDC	I-II	3A	T3N2M0	+	+	-	-	Deceased	23	High
24	F	36	Breast tumor	IDC	II	3C	T2N3M0	-	-	+	+	Deceased	15	Low

25	F	54	Breast tumor	IDC	II	2A	T2N0M0	+	+	-	-	Deceased	60	Low
26	F	63	Breast tumor	IDC	II-III	2B	T2N1M0	+	+	-	-	Deceased	110	Low
27	F	29	Breast tumor	IDC	II	3A	T2N2M0	-	+	-	-	Deceased	78	Low
28	F	48	Breast tumor	IDC	II-III	2B	T2N1M0	NA	NA	NA	NA	Deceased	19	Low
29	F	82	Breast tumor	IDC	II	3A	T3N2M0	-	-	-	-	Deceased	2	Low
30	F	52	Breast tumor	IDC	II	3A	T2N2M0	-	-	+	-	Deceased	110	Low
31	F	31	Breast tumor	IDC	II	3A	T3N2M0	-	-	-	-	Deceased	4	Low
32	F	54	Breast tumor	IDC	III	3A	T2N2M0	+	+	-	-	Deceased	4	High
33	F	71	Breast tumor	IDC	II	2A	T2N0M0	-	-	-	-	Deceased	62	Low
34	F	37	Breast tumor	IDC	II-III	1	T1N0M0	+	-	-	-	Deceased	85	Low
35	F	64	Breast tumor	IDC	II	2A	T2N0M0	-	-	+	+	Deceased	68	Low
36	F	52	Breast tumor	IDC	II	3C	T2N3M0	+	-	+	+	Deceased	61	Low
37	F	73	Breast tumor	IDC	II	3A	T1N2M0	+	+	+	+	Deceased	79	Low
38	F	56	Breast tumor	IDC with ILC	II	3A	T2N2M0	+	+	-	-	Deceased	80	Low
39	F	72	Breast tumor	IDC	II	2A	T1N1M0	-	-	-	-	Deceased	46	Low
40	F	54	Breast tumor	IDC	II	3A	T3N2M0	+	+	+	+	Deceased	9	High
41	F	75	Breast tumor	IDC	II	2B	T2N1M0	+	+	-	-	Deceased	59	Low

Appendix Table S2. Association analysis between JNK activity in TMA samples and clinical parameters. TMA samples used for Kaplan-Meier analysis (Figure 1A) were tested for potential association between p-JNK expression in cancer cells and different clinical variables, such as age at diagnosis, stage, HR status, HER2 status and survival. HR, hormone receptors; ER, estrogen receptor; PR, progesterone receptor; HER2, human epidermal growth factor receptor 2.

		Patient samples			
		**p-JNK high n (%)**	**p-JNK low n (%)**	**Total**	**p-JNK high vs. p-JNK low**
Age (years)	<40	2 (20)	5 (16.1)	7 (17.1)	
	>40	8 (80)	26 (83.9)	34 (82.9)	*P* = 0.6688
Stage	1 or 2	3 (30)	17 (54.8)	20 (48.8)	
	3	7 (70)	14 (45.2)	21 (51.2)	*P* = 0.2017
HR status *	ER+ or PR+	6 (66.7)	16 (55.2)	22 (53.7)	
	ER- and PR-	3 (33.3)	13 (44.8)	16 (39)	*P* = 0.7399
HER2 status *	HER2+	2 (22.2)	10 (34.5)	12 (29.3)	
	HER2-	7 (77.8)	19 (65.5)	26 (63.4)	*P* = 0.7276
Survival (months)	<50	9 (90)	9 (29.1)	18 (43.9)	
	>50	1 (10)	22 (71)	23 (56.1)	*P* = 0.0001

Patient samples, n = 41.

* Three samples did not have information on receptor status, n = 38

P values were determined by a Binomial test.

Appendix Table S3. *P* values for all figures.

**Panels**	***P* values**
Figure 1A	= 0.0022
Figure 1C	= 0.0259
Figure 1D	= 0.0373
Figure 1F	= 0.0025
Figure 1H	*MDA231-LM2*: < 0.0001<br>*SUM159-LM1*: = 0.0012
Figure 1I	*MDA231-LM2*: < 0.0001<br>*SUM159-LM1*: = 0.0364
Figure 1L	= 0.0047
Figure 2C	= 0.0539 (1,000 permutations)
Figure 2D	< 0.0002 (*P* = 0 with 5,000 permutations)
Figure 2E	JNKi vs Vehicle:<br>a) = 0.0002 (5,000 permutations)<br>b) = 0.0024 (5,000 permutations)<br>c) < 0.0002 (*P* = 0 with 5,000 permutations)<br>d) = 0.0032 (5,000 permutations)<br>MKK7-JNK vs Control:<br>a) = 0.0435 (5,000 permutations)<br>b) = 0.0106 (5,000 permutations)<br>c) = 0.0045 (5,000 permutations)<br>d) = 0.0121(5,000 permutations)
Figure 2G	< 0.0001
Figure 3B	< 0.0002 (*P* = 0 with 5,000 permutations)
Figure 3D	= 0.0017 (1000 permutations)
Figure 3E	a) < 0.001 (*P* = 0 with 1,000 permutations)<br>b) = 0.0308 (1,000 permutations)<br>c) = 0.1067 (1,000 permutations)<br>d) = 0.0221 (1,000 permutations)
Figure 3F	Control vs MKK7-JNK: < 0.0001<br>MKK7-JNK vs MKK7-JNK(mut): = 0.0011
Figure 3G	< 0.0001
Figure 3I	< 0.0001 (for all patient samples)
Figure 3M	= 0.0002
Figure 4C	Vehicle vs JNKi (paired tests)<br>*SPP1:* = 0.0079<br>*TNC*: = 0.0002
Figure 4G	< 0.0001 (for both *SPP1* vs *JUN* and *TNC* vs *JUN* correlations)
Figure 4H	Control vs SPP1 def. (1): = 0.0104<br>Control vs SPP1 def. (2): = 0.0215
Figure 4J	Control vs shTNC (1): = 0.0019<br>Control vs shTNC (2): = 0.0401
Figure 4L	= 0.001

Figure 5C	a) < 0.001 (*P* = 0 with 1,000 permutations)<br>b) = 0.1283 (1,000 permutations)<br>c) = 0.0468 (1,000 permutations)<br>d) < 0.001 (*P* = 0 with 1,000 permutations)
Figure 5E	< 0.0001
Figure 6B	*MDA231-LM2*: < 0.0001 (for SPP1 def. + PAX vs any of the other groups)<br>*SUM159-LM1*: < 0.0001 (for SPP1 def. + PAX vs any of the other groups)
Figure 6C	*MDA231-LM2*<br>Control + Vehicle vs SPP1 def. + Vehicle: < 0.0001<br>Control + Vehicle vs SPP1 def. + PAX: < 0.0001<br>Control + PAX vs SPP1 def. + PAX: < 0.0001<br>*SUM159-LM1*<br>Control + Vehicle vs SPP1 def. + Vehicle: = 0.0426<br>Control + Vehicle vs SPP1 def. + PAX: = 0.0210<br>Control + PAX vs SPP1 def. + PAX: = 0.0547
Figure 6D	Control + AC vs SPP1 def. + AC: = 0.0308
Figure 6E	Control + Vehicle vs SPP1 def. + Vehicle: < 0.0001<br>Control + Vehicle vs SPP1 def. + AC: < 0.0001<br>Control + AC vs SPP1 def. + AC: < 0.0001
Figure 6H	Control + PAX vs shTNC + PAX: < 0.0001
Figure 6I	Control + Vehicle vs shTNC + Vehicle: = 0.0002;<br>Control + Vehicle vs shTNC + PAX: < 0.0001<br>Control + PAX vs shTNC + PAX: < 0.0001
Figure 7B	*MDA231-LM2*<br>Vehicle vs PAX: = 0.0004<br>Vehicle vs JNKi: = 0.0005<br>PAX vs JNKi + PAX: = 0.0002<br>JNKi vs JNKi + PAX: < 0.0001<br>*SUM159-LM1*<br>Vehicle vs PAX: = 0.0009<br>Vehicle vs JNKi: = 0.0001<br>PAX vs JNKi + PAX: = 0.0011<br>JNKi vs JNKi + PAX: = 0.0004
Figure 7C	*MDA231-LM2*<br>JNKi vs JNKi + PAX: = 0.0349<br>PAX vs JNKi + PAX: = 0.0153<br>*SUM159-LM1*<br>JNKi vs JNKi + PAX: = 0.0108<br>PAX vs JNKi + PAX: = 0.0325
Figure 7E	Vehicle vs JNKi: = 0.0022<br>Vehicle vs PAX: = 0.0238<br>Vehicle vs JNKi + PAX: = 0.0022<br>JNKi vs JNKi + PAX: = 0.0411
Figure 7F	= 0.0017 (5,000 permutations)
Figure 7G	= 0.0249
Figure 7H	= 0.0227

Figure EV1E	= 0.0003
Figure EV2A	= 0.0117
Figure EV2B	< 0.0001
Figure EV2C	< 0.0001
Figure EV2E	*MDA231-LM2*<br>Vehicle vs MKK7-JNK: < 0.0001<br>MKK7-JNK vs MKK7-JNK(mut): = 0.0003<br>*SUM159-LM1*<br>Vehicle vs MKK7-JNK: < 0.0001<br>MKK7-JNK vs MKK7-JNK(mut): < 0.0001
Figure EV3D	*SPP1:* = 0.0318; *TNC:* = 0.0350 (paired tests)
Figure EV4A	< 0.0001
Figure EV4B	*SPP1:* = 0.0006; *TNC:* < 0.0001
Figure EV5A	< 0.001 (*P* = 0 with 1,000 permutations)
Figure EV5B	< 0.0002 (*P* = 0 with 5,000 permutations)
Appendix Figure S5	< 0.001 (*P* = 0 with 1,000 permutations)
Appendix Figure S7B	Control vs SPP1 def. 1: = 0.9852<br>Control vs SPP1 def. 2: = 0.5391

APPENDIX SUPPLEMENTARY METHODS

Cell culture

M199 medium was supplemented with 2.5% vol/vol FBS, 10 μg/ml insulin, 0.5 μg/ml hydrocortisone, 20 ng/ml epidermal growth factor (EGF, Sigma-Aldrich), 100 ng/ml cholera toxin (Sigma-Aldrich), 0.5 μg/ml amphotericin B, 2 mM l-Glutamine (Sigma-Aldrich), 50 IU/ml penicillin and 50 μg/ml streptomycin. Modified M87 medium was composed of DMEM/F12 + Glutamax (Life Technologies) supplemented with 2% vol/vol FBS, 0.7x insulin-transferrin-selenium-x (Life Technologies), 50 IU/ml penicillin, 50 μg/ml streptomycin, 5 ng/ml EGF, 0.3 μg/ml hydrocortisone (Sigma-Aldrich), 0.5 μg/ml cholera toxin, 5 nM Triiodo-L-thyronine (T3) (Sigma-Aldrich), 0.5 nM β-estradiol (Sigma-Aldrich), 5 μM isoproterenol (Sigma-Aldrich), 50 nM ethanolamine (Sigma-Aldrich) and 50 nM phosphorylethanolamine (Sigma-Aldrich).

In vitro proliferation assay

Proliferation of MDA231-LM2 cells was analyzed using a Cell Titer Blue assay (Promega) according to manufacturer's instructions. Briefly, cancer cells were treated with 4 μM JNK inhibitor (CC-401, Santa Cruz Biotechnology) or vehicle control (0.1% DMSO) for 48 hours and seeded onto 96-well plates (with vehicle and CC-401). At indicated time points, cells were incubated with Cell Titer Blue reagent for 3 hours and fluorescence signal (590 nm) analyzed using a SpectraMax M5 microplate reader (Molecular Devices).

Migration assay

Cells were seeded onto 6-well plates and cultured to confluence. We generated approximately 800 μm wide scratches to the culture using a sterile pipette tip. After 20 hour of migration, pictures were acquired under a light microscope (Zeiss Primovert inverted microscope) for analysis. Wound closure was measured using the Fiji distribution of the ImageJ software (Schindelin et al, 2012) (10-50 measurements per condition). Relative migration was determined

as 1/(EMG/CMG); EMG, experimental migration gap, CMG, control migration gap (empty vector or vehicle control).

Oncosphere formation analysis

To analyze oncosphere formation ability, cancer cells were plated onto 96-well ultra-low attachment plates (Corning) in either full oncosphere medium or HuMEC-medium without supplements at a density of 10,000 cells/ml (200 µl per well). Sphere formation was quantified by counting total number of spheres per well with the help of a Zeiss Primovert microscope. Ten wells were quantified per condition.

Generation of SUM159-LM1

Highly metastatic SUM159-LM1 cell line was generated from SUM159 breast cancer cells by *in vivo* selection as described in (Minn et al, 2005). Briefly, 500,000 TGL-labeled SUM159 cells were inoculated into the lungs of NSG mice via injection into the lateral tail vein. Upon detection of bioluminescent signal in the lungs, mice were sacrificed and lungs were collected and disassociated, first mechanically using a scalpel and then enzymatically by treatment with 0.5% collagenase III (Worthington Biochemical) and 1% dispase II (Life Technologies) in PBS for 1 hour at 37°C. Then cells were washed twice with PBS, pelleted, resuspended in trypsin and incubated for 5 minutes at 37°C. Following trypsin treatment, cells were washed in PBS and plated into adhesive flasks with the appropriate cell culture medium.

Western blot

Cells were lysed with RIPA buffer supplemented with 1x HALT protease and phosphatase inhibitor cocktail and 1x EDTA (both from Thermo Scientific). Protein concentrations were determined using the BCA Protein Assay Kit (Thermo Scientific). Proteins were separated using 4-12% Criterion™ XT Bis-Tris protein gels in the Tris/Glycine/SDS system (Bio-Rad) an transferred to Trans-Blot® Turbo™ Midi PVDF membranes using the Trans-Blot® Turbo™ Transfer System (Bio-Rad). PVDF membranes were then immunoblotted with the indicated

antibodies and luminescence from HRP-conjugated secondary antibodies was detected by exposing the membranes to X-ray films (Fujifilm) or using a ChemiDoc imaging system (Bio-Rad) upon incubation with Clarity Western ECL Substrate (Bio-Rad). For the quantification of p-JNK, JNK, p-c-Jun and c-Jun relative protein levels in normal mammary cells and cancer cells from patient samples, we measured mean grey pixel density of each band and background areas using ImageJ. We then normalized background-corrected measurements to the pixel density values of the corresponding tubulin bands. Quantified protein levels were normalized to tubulin and drawn relative to protein levels in normal mammary epithelial cells (1-7HB2).

Expression of active JNK

Restriction sites in cDNA were introduced by PCR using the following primers:

BstBI-MKK7Jnk1a1-Fw: 5'-CATCTTCGAACGGCAGCCAACATGGAC-3'

XmaI-MKK7Jnk1a1-Rv: 5'- CATCCCCGGGGAGCTCGAGTCACTGCTGC-3'

PCR products and the recipient lentiviral vector pLVX-Puro were digested with BstBI and XmaI in CutSmart buffer (New England Biolabs) in two steps: first, for 2 hours at 37°C and, second, for 1.5 hours at 65°C. Digestion was followed by dephosphorylation with Antarctic Phosphatase (New England Biolabs) and the digested, dephosphorylated DNA fragments were ligated using T4 DNA ligase (New England Biolabs). HEK 293T cells were used to produce lentiviral particles by co-transfecting the cells with the pLVX-Puro vectors containing the Flag-MKK7B2Jnk1a1 or Flag-MKK7B2Jnk1a1(AFP) inserts and the packaging plasmids psPAX2 and pMD2G using Lipofectamine 2000 liposomes (Invitrogen). MDA231-LM2 and SUM159-LM1 cancer cells were infected over night with supernatant containing lentiviral particles in the presence of 8 μg/ml polybrene (Sigma-Aldrich). Infected cells were selected with 2 μg/ml (MDA231-LM2) or 4 μg/ml (SUM159-LM1) puromycin (Invitrogen) for at least 5 days.

mRNA expression analysis

Whole RNA was isolated from cells using the Qiagen RNEasy Kit (Qiagen) and cDNA was generated using the High-Capacity cDNA Reverse Transcription Kit (Applied Biosystems). Gene expression was analyzed using SYBR Green gene expression assay (Applied Biosystems) in the ViiA 7 Real-Time PCR System (Applied Biosystems). The following primer pairs were used:

TNC

Forward: 5'-TAACAGCATCACCCTGGAAT-3'

Reverse: 5'-TCCTTGTCTTCCTTCACAGC-3'

SPP1

Forward: 5'-GATGGCCGAGGTGATAGTGT-3'

Reverse: 5'-GCTTTCCATGTGTGAGGTGA-3'

RPL13A (house-keeping gene)

Forward: 5'-AAGTACCAGGCAGTGACAG-3'

Reverse: 5'-CCTGTTTCCGTAGCCTCATG-3'

Generation of *SPP1* and *TNC* knockdown cells

For SPP1 knockdown, the following mature antisense sequences were used:

Hairpins used in GIPZ vectors

V3LHS_303525: 5'-AGATTTTGACCTCAGTCCA-3'

V3LHS_303526: 5'-ACATCATCAGAGTCGTTCG-3'

Hairpins used in miR-E vectors

shSPP1 (1):

5'-TGCTGTTGACAGTGAGCGCCCCCACAGTAGACACATATGATAGTGAAGCCACAGATGTATCAT ATGTGTCTACTGTGGGGATGCCTACTGCCTCGGA-3'

shSPP1 (2):

5’-TGCTGTTGACAGTGAGCGATCGAACGACTCTGATGATGTATAGTGAAGCCACAGATGTATACATC ATCAGAGTCGTTCGAGTGCCTACTGCCTCGGA-3’.

Non-silencing control:

5’-TGCTGTTGACAGTGAGCGCTCTCGCTTGGGCGAGAGTAAGTAGTGAAGCCACAGATGTACTTACT CTCGCCCAAGCGAGATTGCCTACTGCCTCGGA-3’.

We designed the miR-E shSPP1 oligonucleotides using the shERWOOD algorithm tool (Knott et al, 2014). Oligonucleotides were amplified by PCR using the Q5 High-Fidelity DNA Polymerase (New England Biolabs). The following primers were used for PCR amplification:

miRE-Xho-fw: 5’-TGAACTCGAGAAGGTATATTGCTGTTGACAGTGAGCG-3’

miRE-EcoOligo-rev: 5’-TCTCGAATTCTAGCCCCTTGAAGTCCGAGGCAGTAGGC-3’

PCR products containing shSPP1 and non-silencing miR-Es were subcloned into the StagBFPEP recipient vector via EcoRI-HF and XhoI restriction sites. Lentiviral particles were produced by transfecting HEK293T cells with StagBFPEP-miR-E shSPP1 or StagBFPEP-miR-E shControl (non-silencing miR-E) together with plasmids encoding for the packaging proteins pMD2G and psPAX2 using Lipofectamine 2000 (Invitrogen). Supernatants containing lentiviral particles were then used to infect cancer cells overnight in the presence of 8 µg/ml polybrene (Sigma-Aldrich). Infected cells were selected with 2 µg/ml (MDA231-LM2) or 4 µg/ml (SUM159-LM1) puromycin (Invitrogen) in cell culture medium for 5 days.

For TNC knockdown, the following hairpin sequences were used:

TRCN0000157688:

5’-CCGGCCAGGAATCTTCGACGTGTTTCTCGAGAAACACGTCGAAGATTCCTGGTTTTTTG-3’

TRCN0000154001:

5’-CCGGCCACTGGAAATAACCCTACTTCTCGAGAAGTAGGGTTATTTCCAGTGGTTTTTTG-3’

ChIP-qPCR

Primers for qPCR analysis were designed flanking consensus and tracked AP-1/c-Jun binding sites within SPP1 and TNC promoter regions with the help of the UCSC Genome Browser (GRCh37/hg19 assembly) (Kent et al, 2002). The following primer sequences were used:

SPP1

Primer pair 1:

Forward: 5'-ATAGCGGGTCATTGTTGGGA-3'

Reverse: 5'-TTCCAGCGGGATAGAACACTC-3'

Primer pair 2:

Forward: 5'-CATGCCGACCATAACGCAAG-3'

Reverse: 5'-GGTGCAATGGACTGTGTTCG-3'

TNC

Primer pair 1:

Forward: 5'-CCCCCACAGCCCTTTCTTTA-3'

Reverse: 5'-GAAAGCAAAGGCGCAGCTTA-3'

Primer pair 2:

Forward: 5'-TCACCTAACTTCCTTGAGTGTCG-3'

Reverse: 5'-AGGAGGGTTTGCCAATGGTT-3'

qPCR data was analyzed using the following formula: $2^{(Ct_{sample} – Ct_{input})}$x(input %), where Ct values of immunoprecipitated samples were plotted in relation to the total input.

TUNEL assay

To analyze apoptosis in xenograft mouse models of breast cancer, DNA strand breaks were labeled using the In Situ Cell Death Detection Kit, TMR Red (Roche) following manufacturer's instructions. Briefly, sections from frozen, OCT-embedded xenograft tumors were cut and fixed

with 4% formaldehyde for 20 minutes at room temperature. Sections were washed with PBS for 30 minutes and permeabilised using 0.1% triton X-100 sodium citrate. This was followed by incubation with TUNEL reaction mixture for 1h at 37°C, in the dark, in a humidified chamber. Slides were rinsed in PBS, mounted with a cover slide and TUNEL staining visualized using a Zeiss Cell Observer microscope. TUNEL positive cells in tumor sections were quantified using the Fiji distribution of ImageJ.

Flow cytometry

To analyze apoptosis in vitro, MDA231-LM2 cancer cells were stained with Annexin V coupled to Alexa 647 fluorochrome (BioLegend) and propidium iodide (PI, Sigma-Aldrich), according to manufacturer's instructions. Data was acquired using an LSR Fortessa cytometer (BD Biosciences) and analysis performed using FlowJo X software version 10.0.7r2 (FlowJo LCC).

Supplementary References

Chiaretti S, Li X, Gentleman R, Vitale A, Vignetti M, Mandelli F, Ritz J, Foa R (2004) Gene expression profile of adult T-cell acute lymphocytic leukemia identifies distinct subsets of patients with different response to therapy and survival. *Blood* 103**:** 2771-2778

Creighton CJ, Massarweh S, Huang S, Tsimelzon A, Hilsenbeck SG, Osborne CK, Shou J, Malorni L, Schiff R (2008) Development of resistance to targeted therapies transforms the clinically associated molecular profile subtype of breast tumor xenografts. *Cancer Research* 68**:** 7493-7501

Hann CL, Daniel VC, Sugar EA, Dobromilskaya I, Murphy SC, Cope L, Lin X, Hierman JS, Wilburn DL, Watkins DN et al (2008) Therapeutic efficacy of ABT-737, a selective inhibitor of BCL-2, in small cell lung cancer. *Cancer Research* 68**:** 2321-2328

Huang F, Reeves K, Han X, Fairchild C, Platero S, Wong TW, Lee F, Shaw P, Clark E (2007) Identification of candidate molecular markers predicting sensitivity in solid tumors to dasatinib: rationale for patient selection. *Cancer Research* 67**:** 2226-2238

Huper G, Marks JR (2007) Isogenic normal basal and luminal mammary epithelial isolated by a novel method show a differential response to ionizing radiation. *Cancer Research* 67**:** 2990-3001

Kang HC, Kim IJ, Park JH, Shin Y, Ku JL, Jung MS, Yoo BC, Kim HK, Park JG (2004) Identification of genes with differential expression in acquired drug-resistant gastric cancer cells using high-density oligonucleotide microarrays. *Clinical Cancer Research* : an official journal of the American Association for Cancer Research 10**:** 272-284

Kent WJ, Sugnet CW, Furey TS, Roskin KM, Pringle TH, Zahler AM, Haussler D (2002) The human genome browser at UCSC. *Genome Research* 12**:** 996-1006

Knott SRV, Maceli A, Erard N, Chang K, Marran K, Zhou X, Gordon A, Demerdash OE, Wagenblast E, Kim S et al (2014) A computational algorithm to predict shRNA potency. *Molecular Cell* 56**:** 796-807

Kyng KJ, May A, Stevnsner T, Becker KG, Kolvra S, Bohr VA (2005) Gene expression responses to DNA damage are altered in human aging and in Werner Syndrome. *Oncogene* 24**:** 5026-5042

Li J, Wood WH, 3rd, Becker KG, Weeraratna AT, Morin PJ (2007) Gene expression response to cisplatin treatment in drug-sensitive and drug-resistant ovarian cancer cells. *Oncogene* 26**:** 2860-2872

Massarweh S, Osborne CK, Creighton CJ, Qin L, Tsimelzon A, Huang S, Weiss H, Rimawi M, Schiff R (2008) Tamoxifen resistance in breast tumors is driven by growth factor receptor signaling with repression of classic estrogen receptor genomic function. *Cancer Research* 68**:** 826-833

McDowell SA, Gammon K, Zingarelli B, Bachurski CJ, Aronow BJ, Prows DR, Leikauf GD (2003) Inhibition of nitric oxide restores surfactant gene expression following nickel-induced acute lung injury. *American Journal of Respiratory Cell and Molecular Biology* 28**:** 188-198

Minn AJ, Gupta GP, Siegel PM, Bos PD, Shu W, Giri DD, Viale A, Olshen AB, Gerald WL, Massagué J (2005) Genes that mediate breast cancer metastasis to lung. *Nature* 436: 518-24.

Naba A, Clauser KR, Hoersch S, Liu H, Carr SA, Hynes RO (2012) The matrisome: in silico definition and in vivo characterization by proteomics of normal and tumor extracellular matrices. *Molecular & Cellular Proteomics* : MCP 11**:** M111 014647

Patterson SG, Wei S, Chen X, Sallman DA, Gilvary DL, Zhong B, Pow-Sang J, Yeatman T, Djeu JY (2006) Novel role of Stat1 in the development of docetaxel resistance in prostate tumor cells. *Oncogene* 25**:** 6113-6122

Riggins RB, Lan JP, Zhu Y, Klimach U, Zwart A, Cavalli LR, Haddad BR, Chen L, Gong T, Xuan J et al (2008) ERRgamma mediates tamoxifen resistance in novel models of invasive lobular breast cancer. *Cancer Research* 68**:** 8908-8917

Roy S, Patel D, Khanna S, Gordillo GM, Biswas S, Friedman A, Sen CK (2007) Transcriptome-wide analysis of blood vessels laser captured from human skin and chronic wound-edge tissue. *Proceedings of the National Academy of Sciences of the United States of America* 104**:** 14472-14477

Schindelin J, Arganda-Carreras I, Frise E, Kaynig V, Longair M, Pietzsch T, Preibisch S, Rueden C, Saalfeld S, Schmid B et al (2012) Fiji: an open-source platform for biological-image analysis. *Nature Methods* 9**:** 676-682

Theilgaard-Monch K, Knudsen S, Follin P, Borregaard N (2004) The transcriptional activation program of human neutrophils in skin lesions supports their important role in wound healing. *J Immunol* 172**:** 7684-7693
