# Supplementary material for: Stress signaling in breast cancer cells induces matrix components that promote chemoresistant metastasis
Source: EMBO Mol Med. 2018 Sep 6;10(10):e9003. doi: 10.15252/emmm.201809003 (PMC6180299; doi:10.15252/emmm.201809003)
Supplement: Supplementary file 2 — Expanded View Figures PDF [file EMMM-10-e9003-s002.pdf]

Expanded View Figures

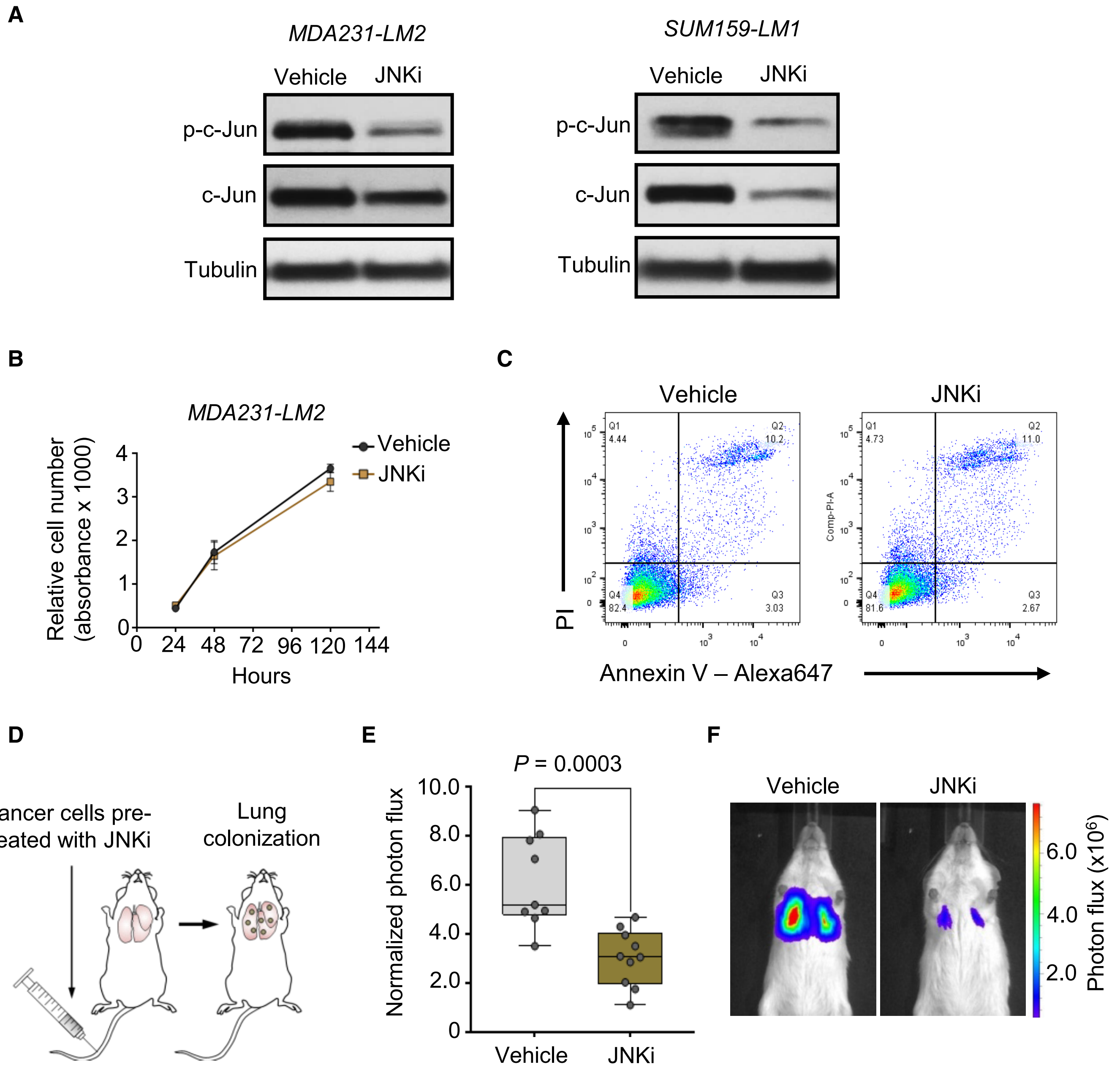

Figure EV1. Inhibition of JNK signaling in breast cancer cells and lung colonization in a xenograft mouse model.

A Western blot analysis of p-c-Jun and c-Jun in MDA231-LM2 and SUM159-LM1 breast cancer cells treated with vehicle (DMSO) or 4 μM JNK inhibitor (JNKi). Tubulin was used as a loading control.

B Cell growth of MDA231-LM2 cells treated with JNK inhibitor. Data points depict mean Cell Titer Blue absorbance values from triplicate experiments ± SD.

C FACS analysis of apoptosis in JNKi-treated MDA231-LM2 cells by Annexin V and propidium iodide (PI) staining.

D Schematic of experimental setup used to analyze the functional role of JNK signaling in breast cancer cells during lung colonization. Cancer cells were pre-treated with JNKi 48 h before injection. Mice were not treated with JNKi.

E Lung colonization in NSG mice intravenously injected with MDA231-LM2 cancer cells pre-treated with 4 μM JNKi. Boxes depict median with upper and lower quartiles. Whiskers show maximum and minimum. Vehicle $n = 9$ mice; JNKi $n = 10$ mice. P-value was determined by two-tailed Mann–Whitney test.

F Representative bioluminescence example of mice with lung metastases.

Source data are available online for this figure.

© 2018 The Authors

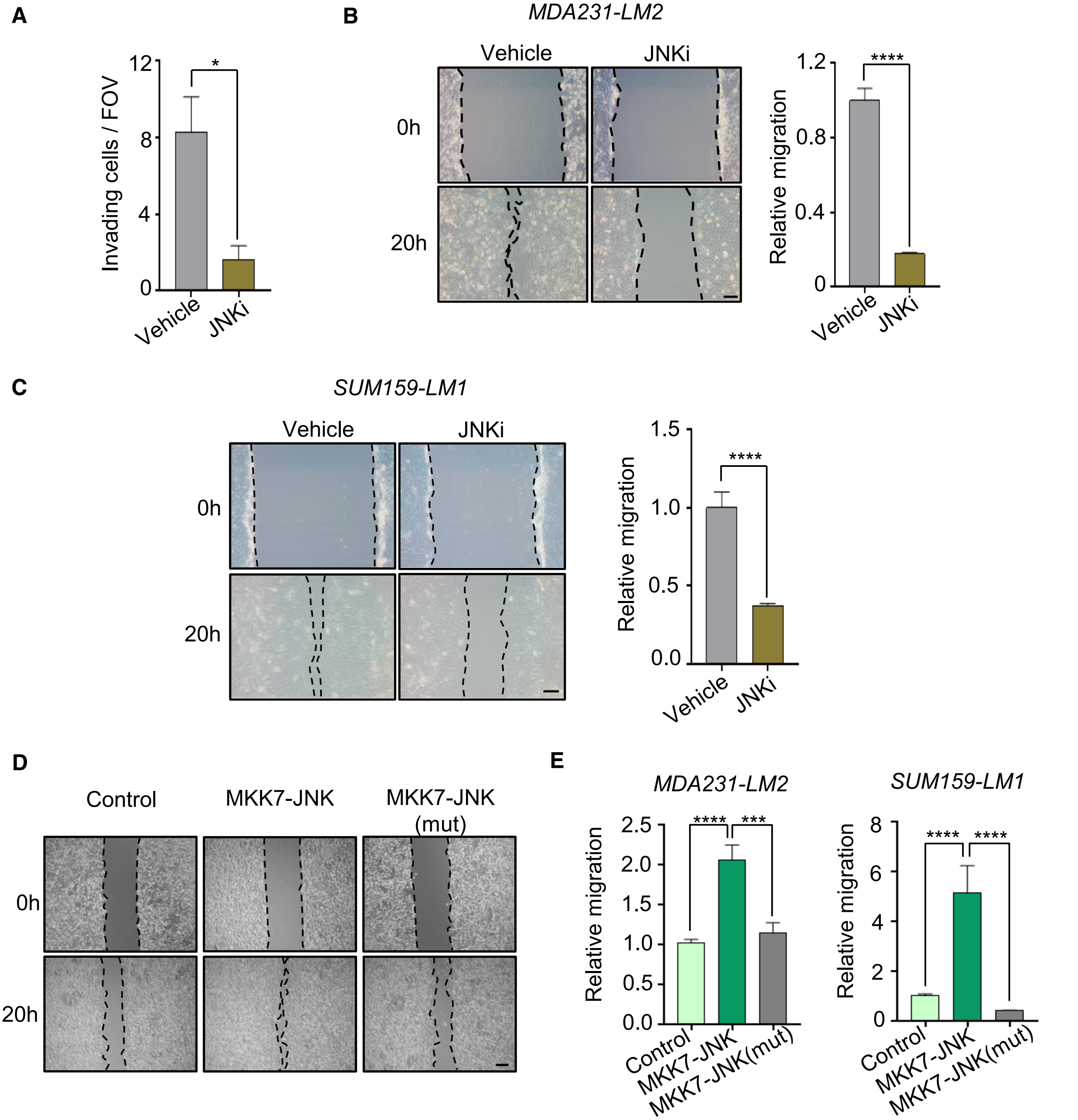

Figure EV2. Role of JNK signaling in cell motility and invasion.

A Quantification of control (vehicle) and JNKi-treated SUM159-LM1 cancer cells invading through Matrigel in a transwell invasion assay. FOV, field of view. Values are means from eight measurements per group ± SEM.

B Migration of MDA231-LM2 cells treated with vehicle or JNKi. Left, examples of cancer cells migrating into scratch wound. Scale bar, 100 μm. Right, quantification of relative migration, indicated by means from 10 measurements per group ± SEM.

C Migration of SUM159-LM1 cells treated with vehicle or JNKi in a scratch assay. Left, representative images from before (0 h) and after 20 h (20 h) of migration. Scale bar, 100 μm. Right, quantification of relative migration of control (vehicle) and JNKi-treated SUM159-LM1 in a scratch assay. Values are means from 10 measurements per group ± SEM.

D Relative migration of MDA231-LM2 cells expressing MKK7-JNK or MKK7-JNK(mut) in a scratch assay at 0 and 20 h (representative images). Scale bar 500 μm.

E Quantification of relative migration in MDA231-LM2 and SUM159-LM1 cancer cells expressing MKK7-JNK or MKK7-JNK(mut) in a scratch assay. Values are means from 10 (MDA231-LM2) or 50 (SUM159-LM1) measurements per group ± SEM.

Data information: Panels (A–C and E), *$P < 0.05$, ***$P < 0.001$, ****$P < 0.0001$. P-values were determined by two-tailed Mann–Whitney test.

© 2018 The Authors

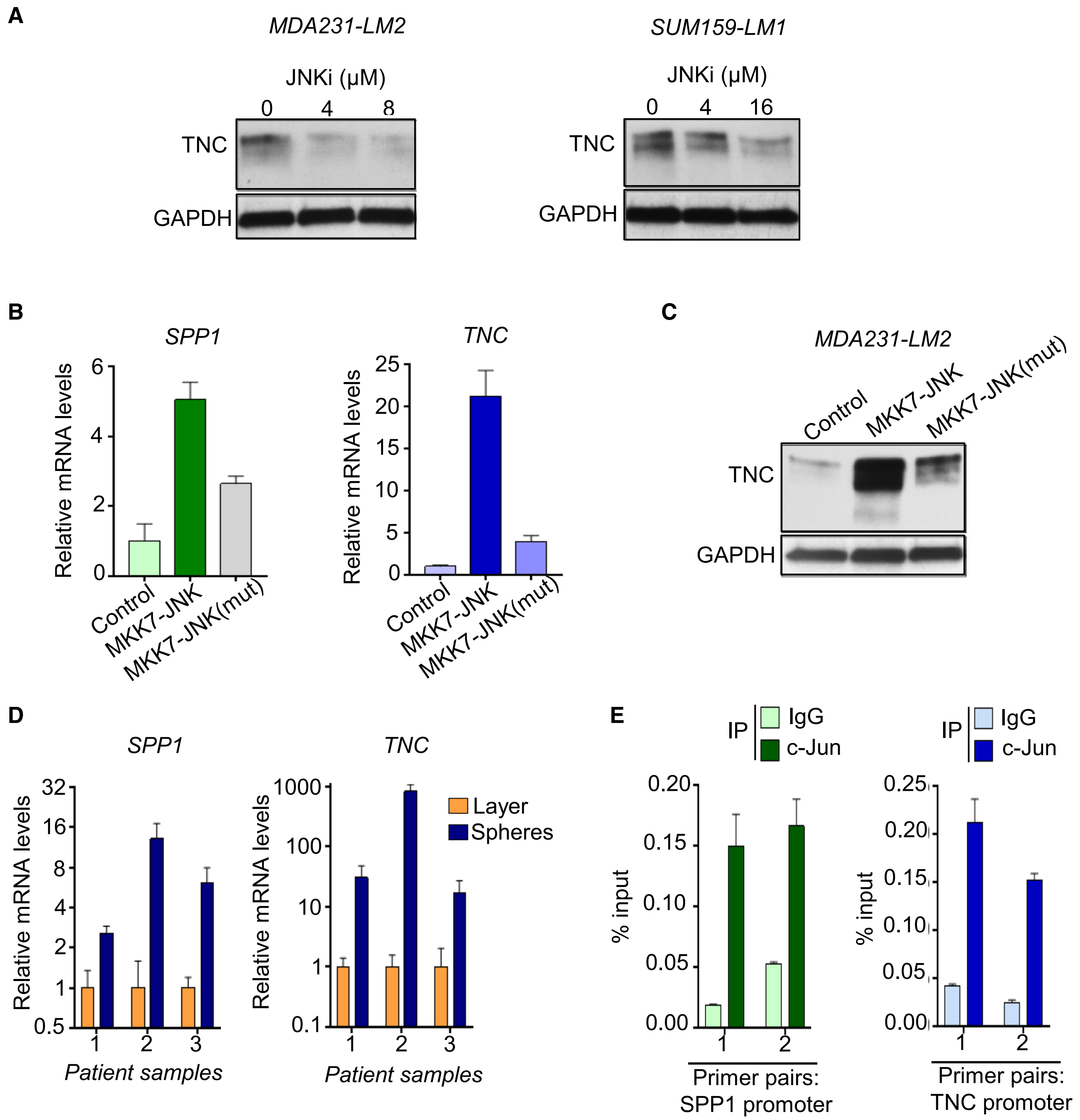

Figure EV3. Analysis of *SPP1* and *TNC* regulation by JNK signaling.

A Western blot analysis of TNC expression in MDA231-LM2 and SUM159-LM1 cancer cells treated with incremental concentrations of JNK inhibitor. Loading control, GAPDH.

B SPP1 and TNC mRNA levels in SUM159-LM1 cells expressing constitutively active JNK (MKK7-JNK) or inactive mutated JNK (MKK7-JNK(mut)). Expression levels were determined using qPCR. Shown are means of triplicates ± SD.

C Western blot analysis of TNC protein levels in MDA231-LM2 cancer cells expressing active JNK or a mutated inactive JNK. GAPDH was used as a loading control.

D SPP1 and TNC expression in patient-derived breast cancer cells grown as oncospheres. Samples 1–2 are ascites samples; sample 3 is a pleural effusion sample. Expression levels were determined using qPCR. Shown are means of triplicates ± SD. The results from all three patient samples were included to calculate significance of gene induction in spheres. *SPP1*, $P = 0.032$; *TNC*, $P = 0.035$. *P*-values were determined by a paired Student's *t*-test.

E ChIP-qPCR analysis of c-Jun binding to *SPP1* and *TNC* promoters in SUM159-LM1 cells. *SPP1* and *TNC* promoter fragments were pulled down by c-Jun or IgG antibodies. Values represent means of qPCR triplicates ± SD.

Source data are available online for this figure.

© 2018 The Authors

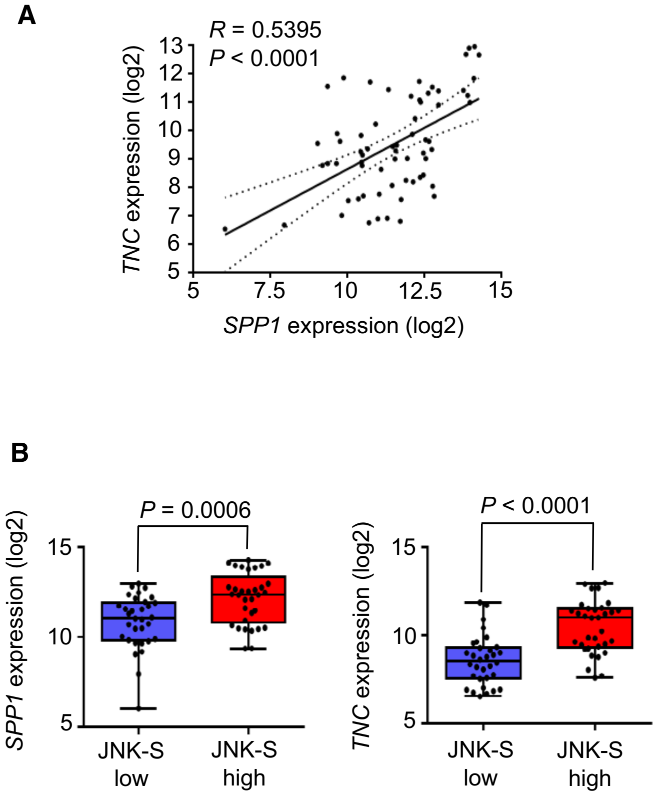

Figure EV4. Correlation analysis of *SPP1*, *TNC*, and JNK-S (JNK signature) in human metastasis samples.

A Correlation of *SPP1* and *TNC* expression in dissected metastatic lesions from breast cancer patients (GSE14020). $N = 65$ patient samples. *P*-value was determined by Student's *t*-test.

B Expression of *SPP1* (left) and *TNC* (right) in metastasis samples from breast cancer patients (GSE14020), classified according to JNK signature score. Values are median with upper and lower quartiles. Whiskers represent maximum and minimum values. JNK-S low $n = 32$ patient samples, JNK-S high $n = 33$ patient samples. *P*-value was determined by a two-tailed Student's *t*-test.

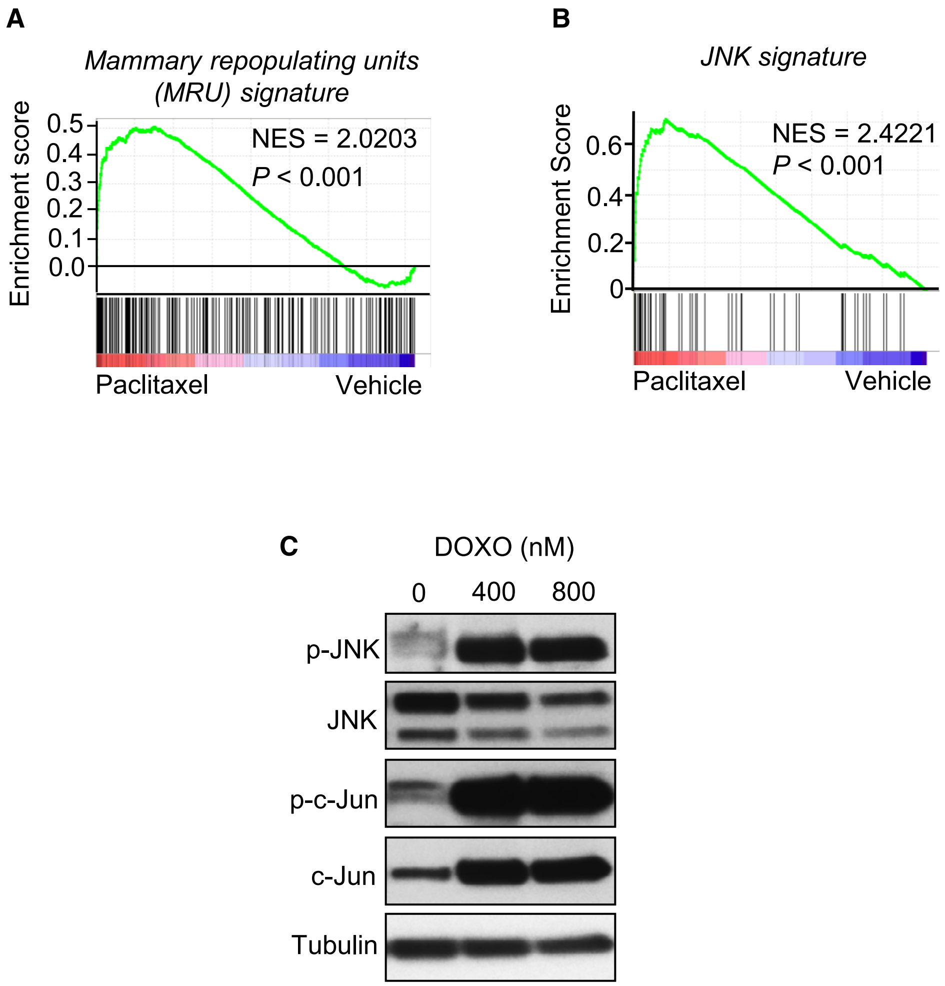

Figure EV5. Expression of stem cell signature and JNK signaling in breast cancer cells treated with chemotherapy.

A GSEA graph showing enrichment of a mammary stem cell signature (mammary repopulating units, MRU) (Stingl *et al*, 2006), in MDA231-LM2 cells treated with paclitaxel (PAX). NES, normalized enrichment score. *P*-value was determined by random permutation test.

B GSEA of JNK signature in the gene expression data set of paclitaxel-treated MDA231-LM2 cells. NES, normalized enrichment score. *P*-value was determined by random permutation test.

C Western blot analysis of p-JNK, JNK, p-c-Jun, and c-Jun in SUM159-LM1 cells treated with incremental concentrations of doxorubicin (DOXO). Tubulin was used as a loading control.

Source data are available online for this figure.

 © 2018 The Authors
