# Supplementary figures and images for: Stress signaling in breast cancer cells induces matrix components that promote chemoresistant metastasis
Source: EMBO Mol Med. 2018 Sep 6;10(10):e9003. doi: 10.15252/emmm.201809003 (PMC6180299; doi:10.15252/emmm.201809003)

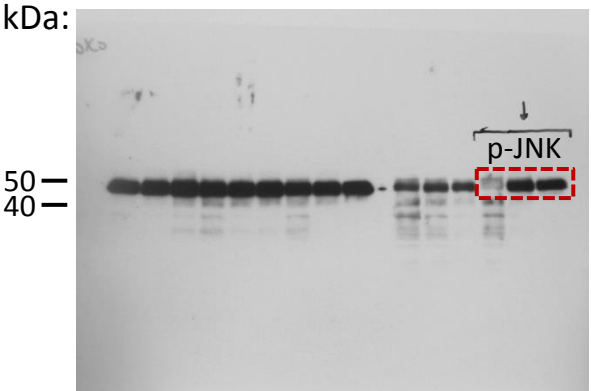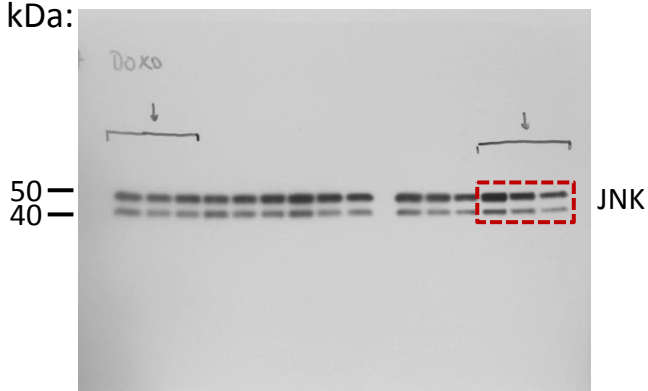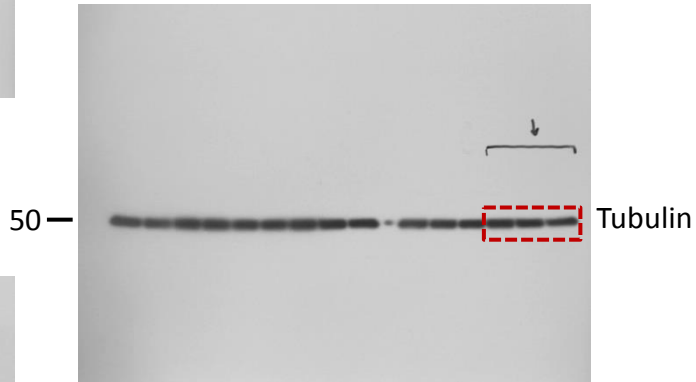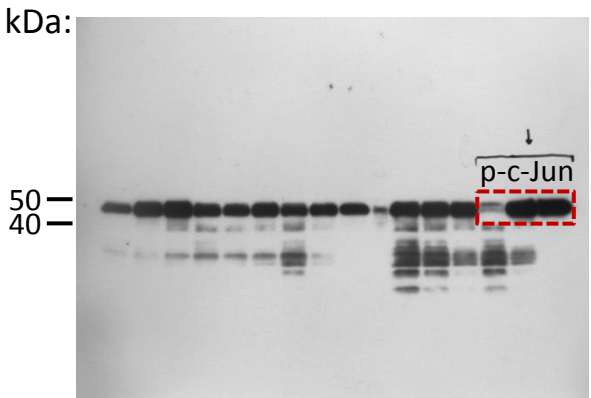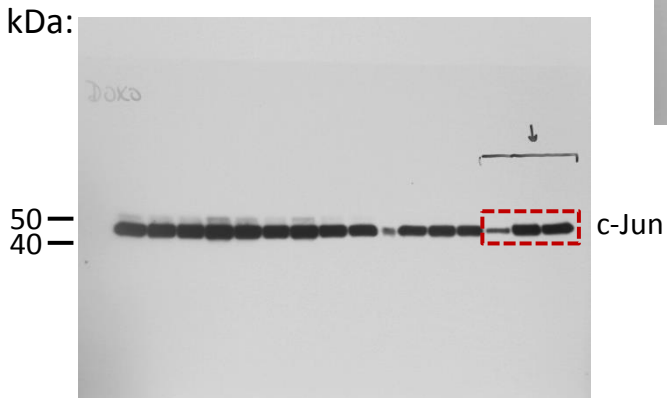

Supplement: Supplementary file 3 — Source Data for Expanded View and Appendix [file EMMM-10-e9003-s005.zip › EMM-2018-09003-V3_Source_Data_FigEV5.pdf]

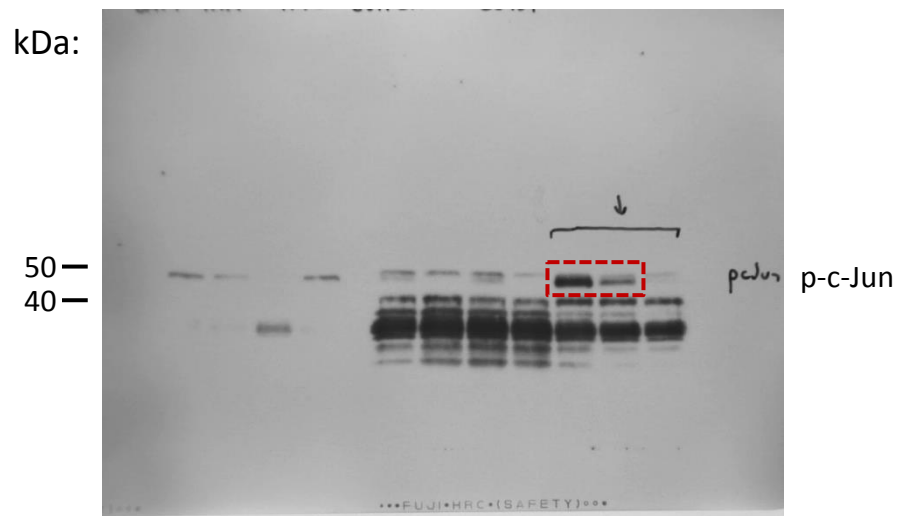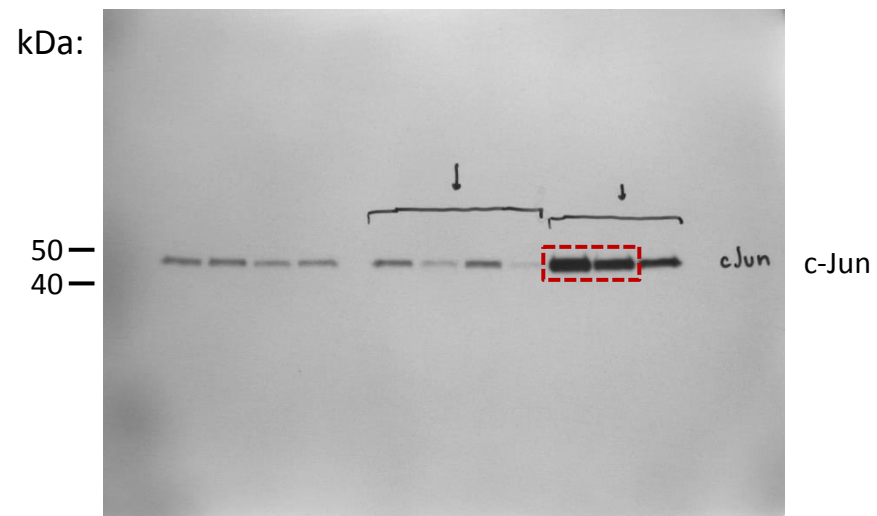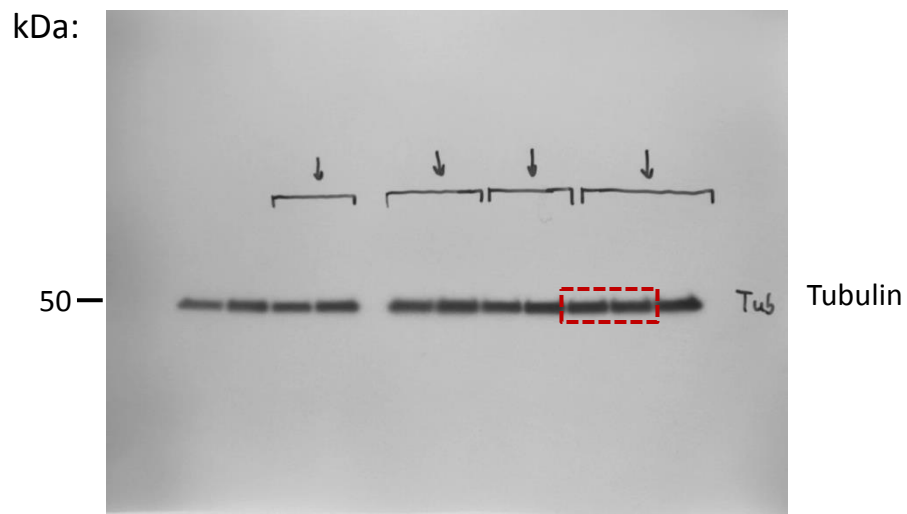

Source Data-EV1A (left panel, MDA231-LM2)

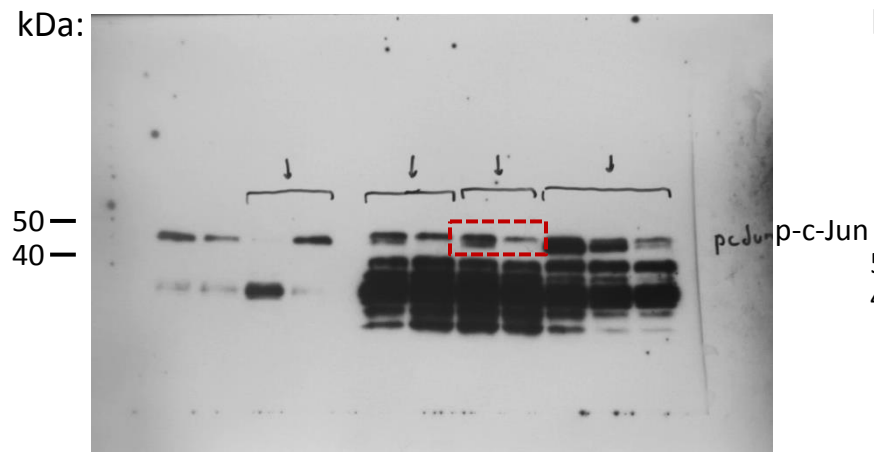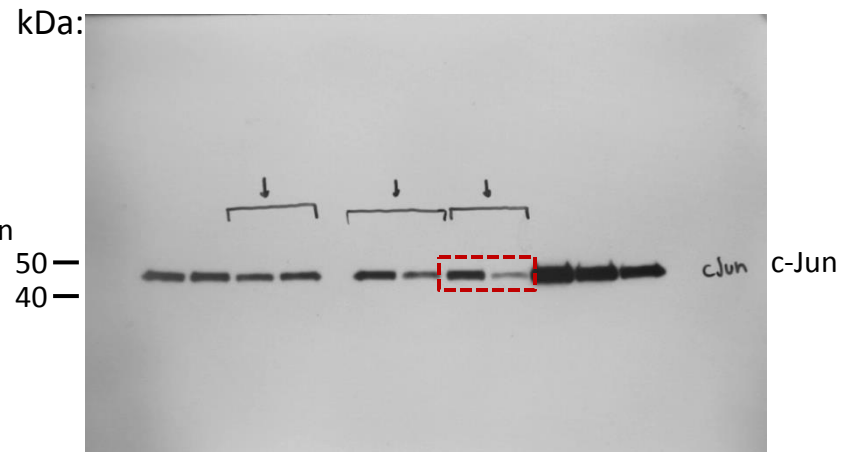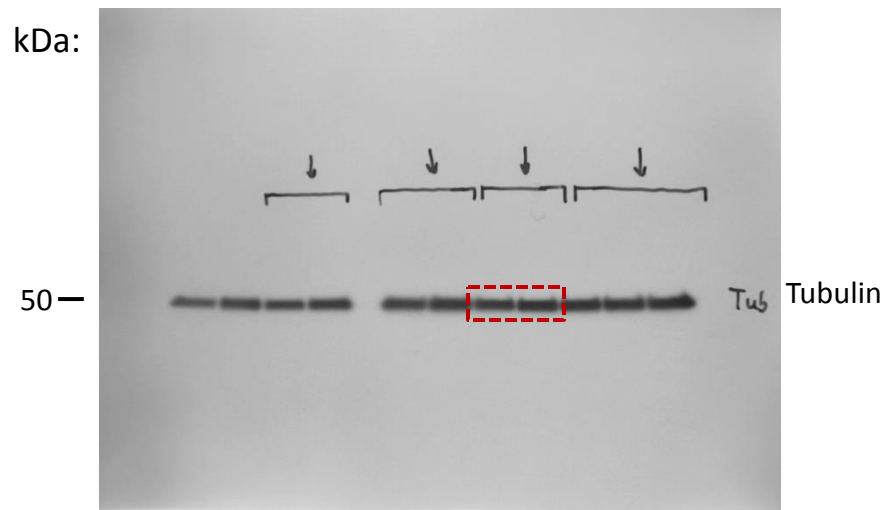

Source Data-EV1A (right panel, SUM159-LM1)

Supplement: Supplementary file 3 — Source Data for Expanded View and Appendix [file EMMM-10-e9003-s005.zip › EMM-2018-09003-V3_Source_Data_FigEV1.pdf]

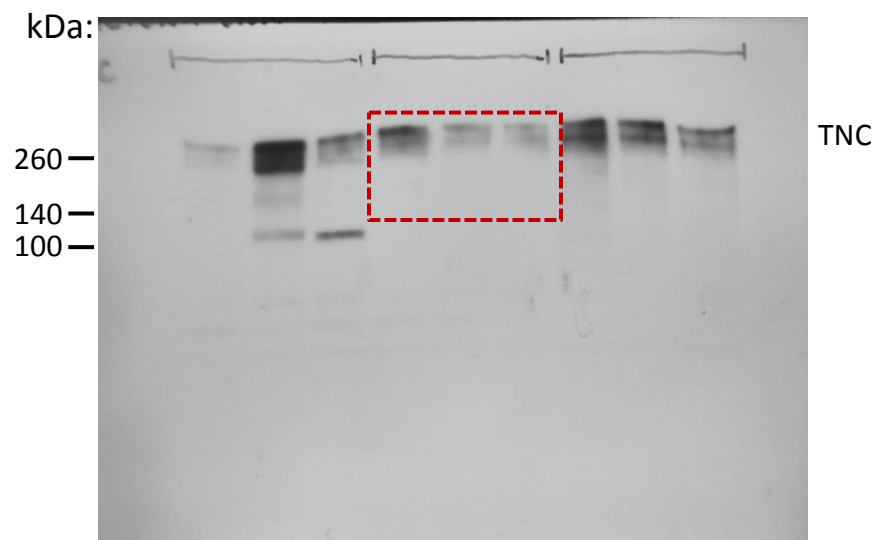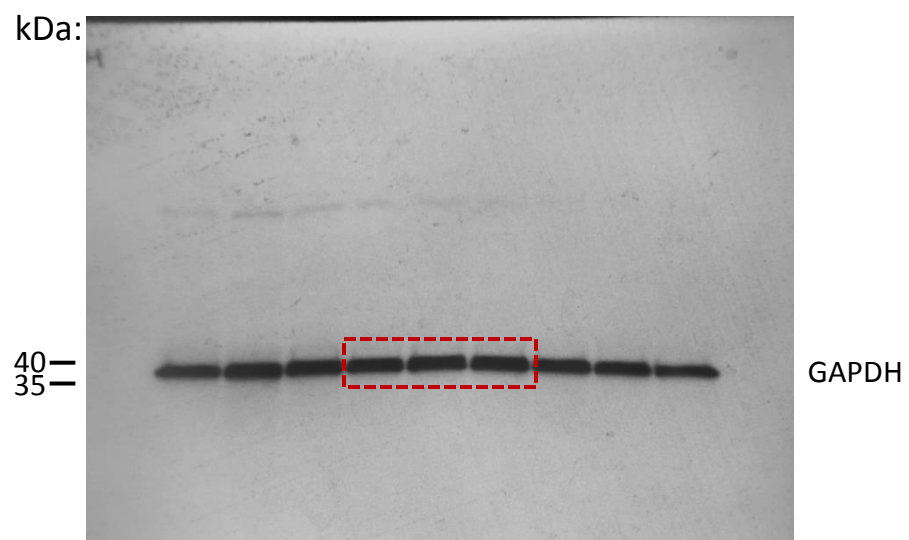

Source Data-EV3A (left panel, MDA231-LM2)

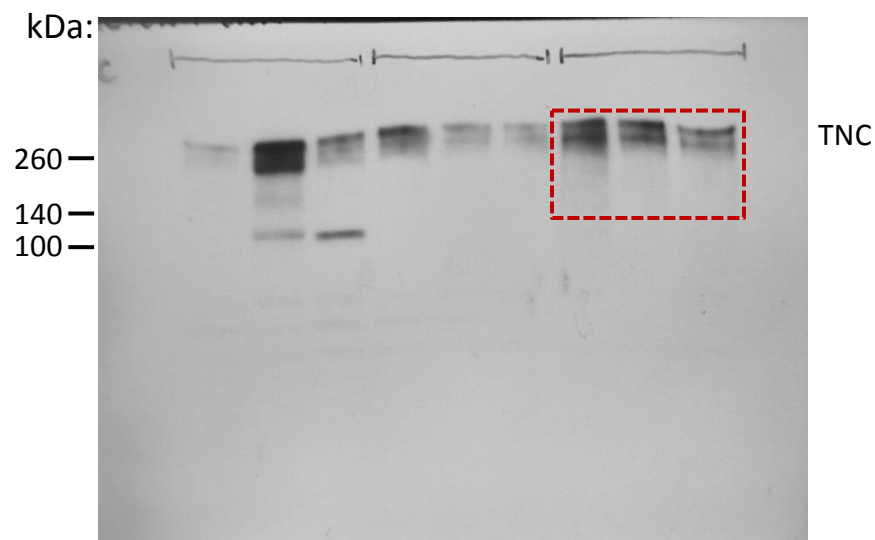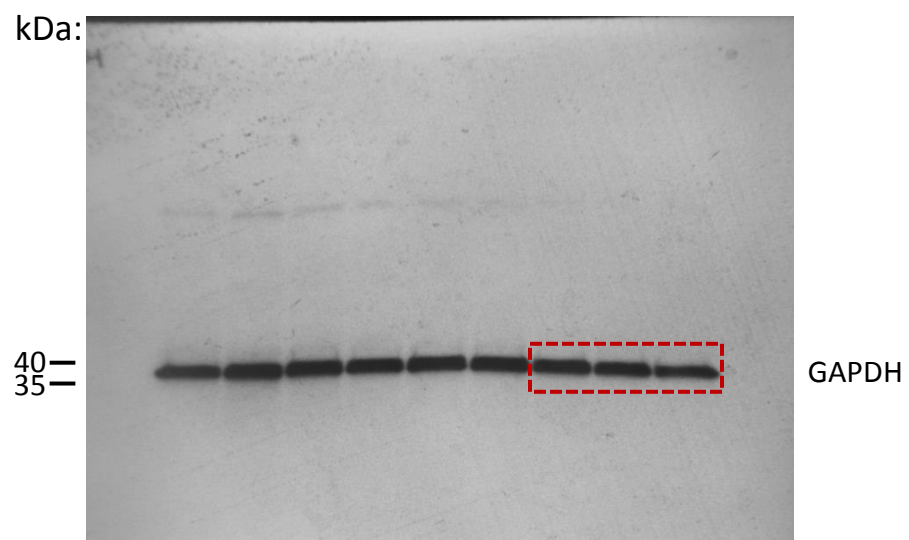

Source Data-EV3A (right panel, SUM159-LM1)

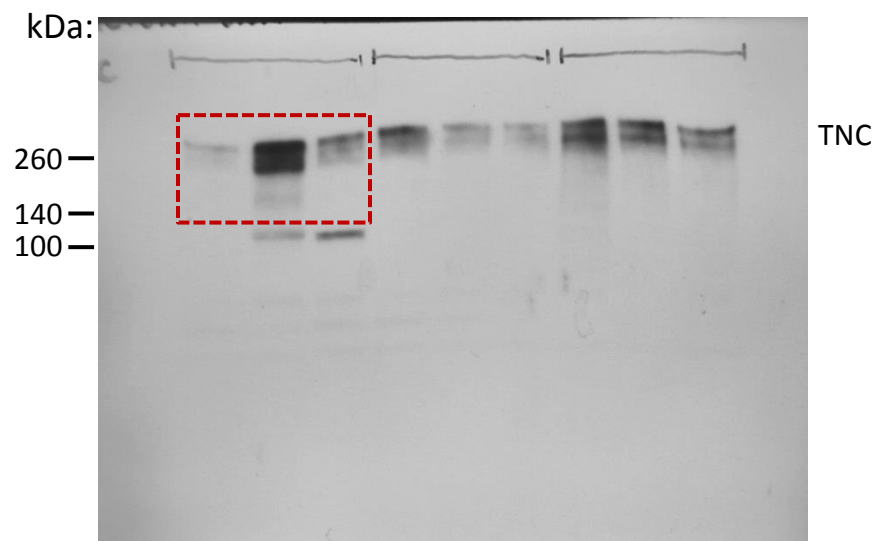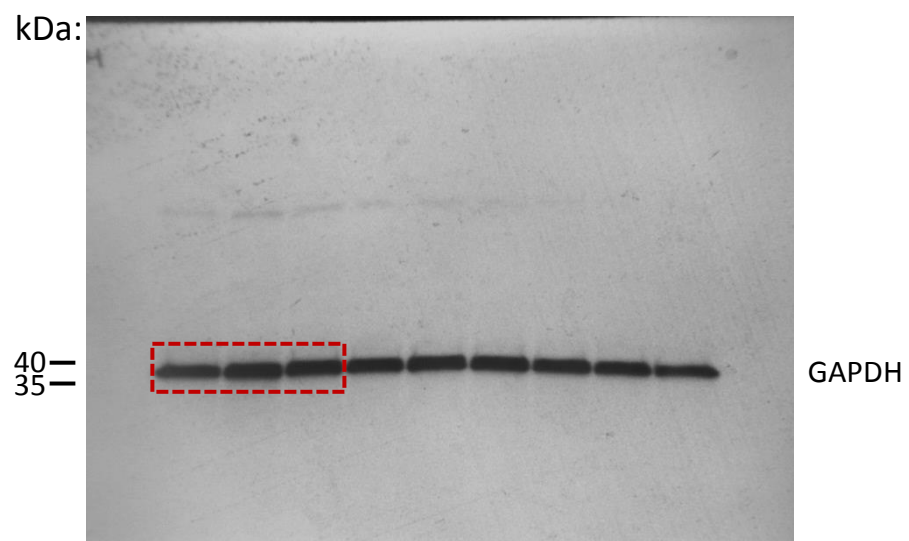

Supplement: Supplementary file 3 — Source Data for Expanded View and Appendix [file EMMM-10-e9003-s005.zip › EMM-2018-09003-V3_Source_Data_FigEV3.pdf]

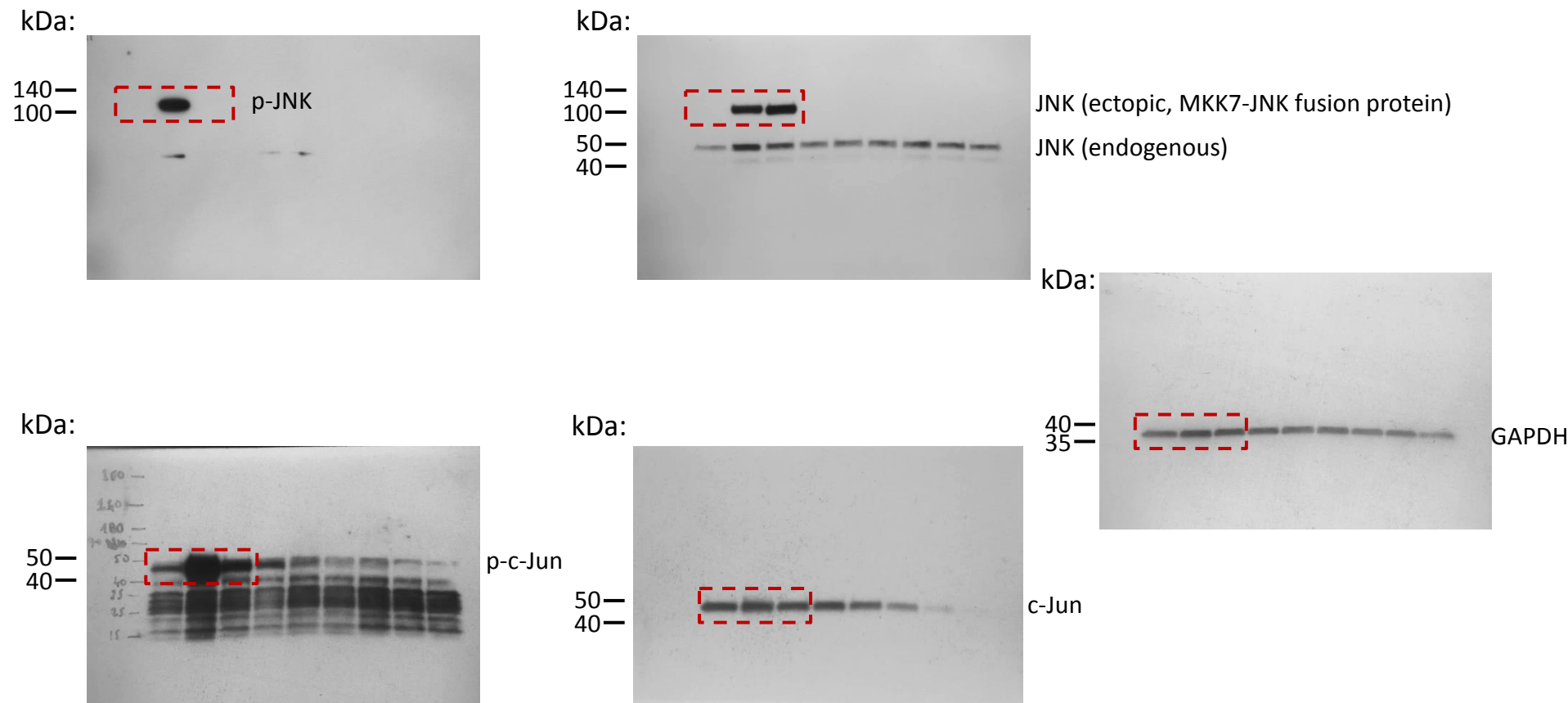

Source Data-Appendix Figure S3A

Supplement: Supplementary file 3 — Source Data for Expanded View and Appendix [file EMMM-10-e9003-s005.zip › Source_Data_for_Appendix_FiguresS3.pdf]

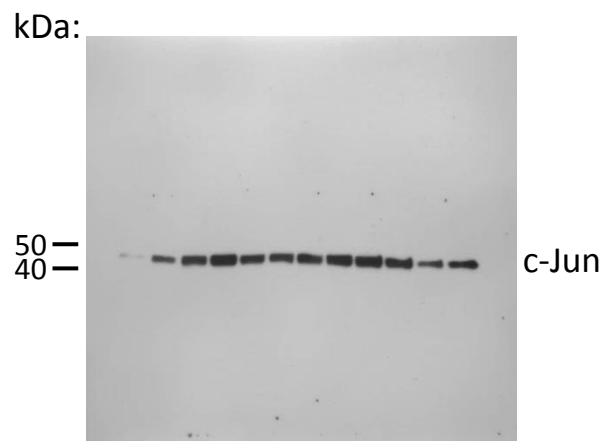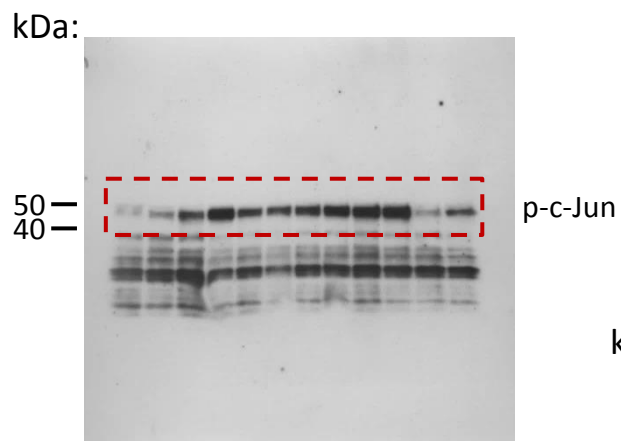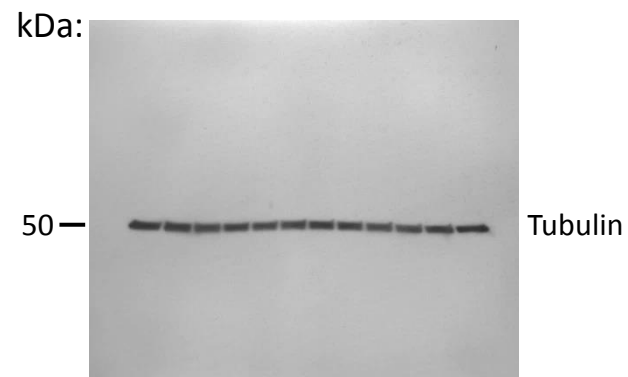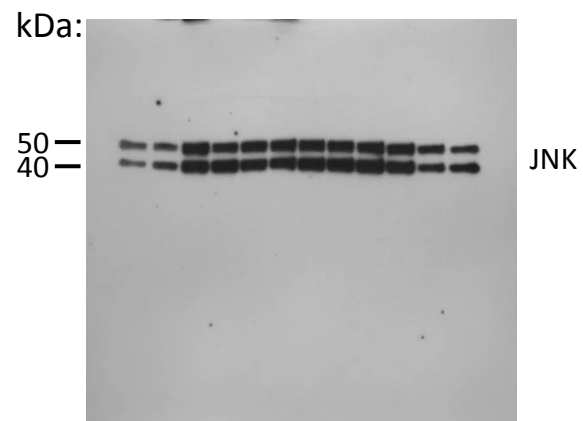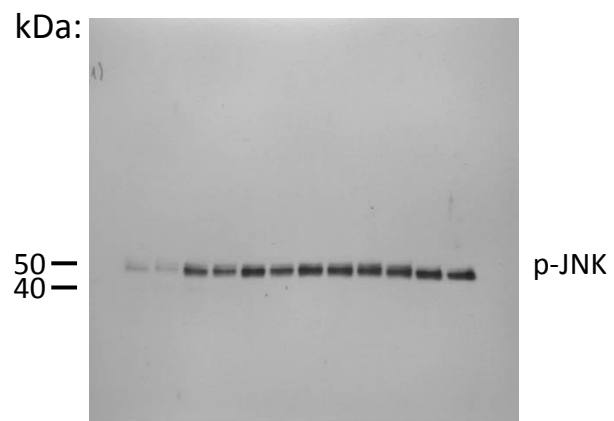

Supplement: Supplementary file 3 — Source Data for Expanded View and Appendix [file EMMM-10-e9003-s005.zip › Source_Data_for_Appendix_FiguresS1.pdf]

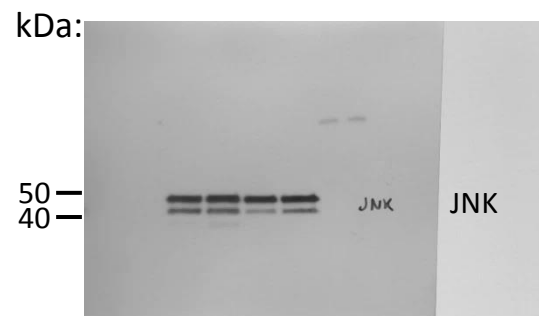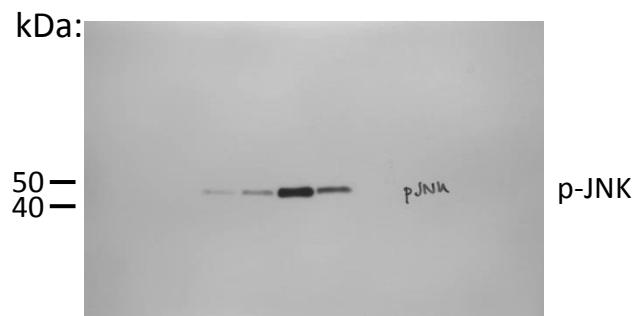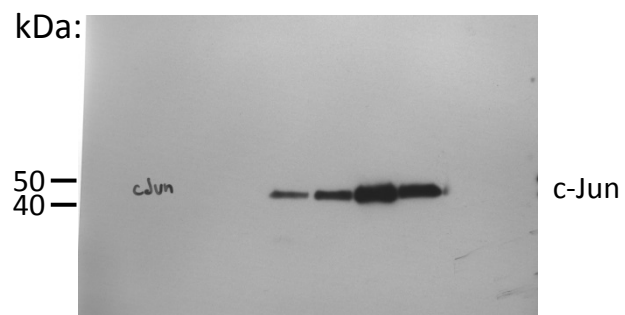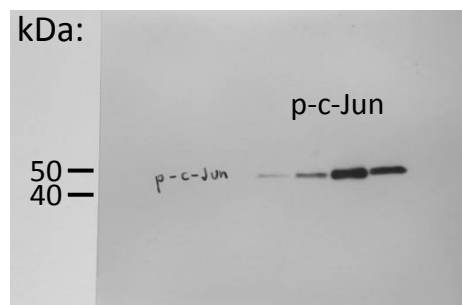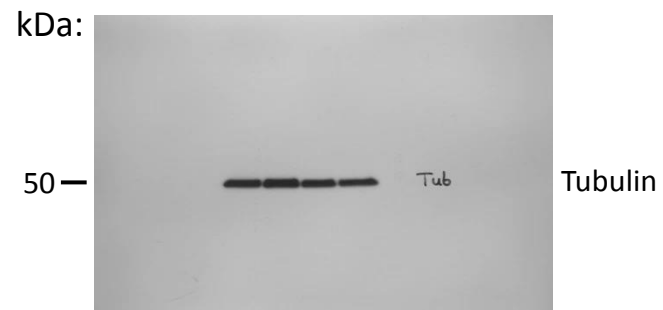

Supplement: Supplementary file 3 — Source Data for Expanded View and Appendix [file EMMM-10-e9003-s005.zip › Source_Data_for_Appendix_FiguresS2.pdf]

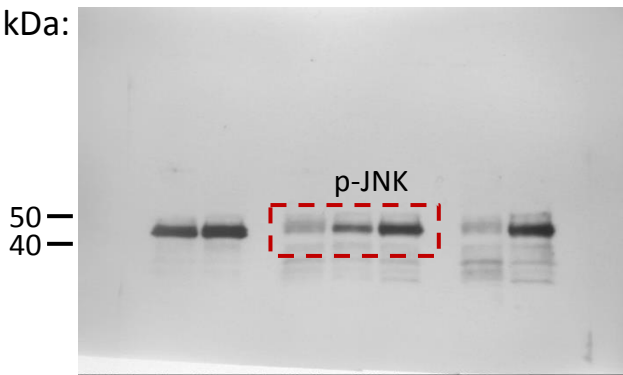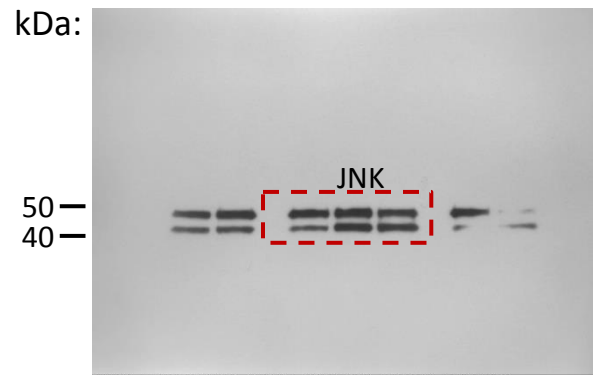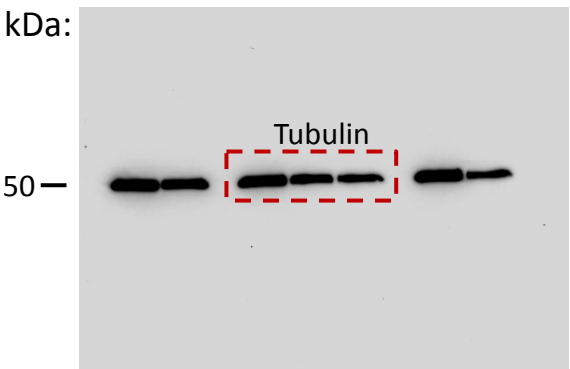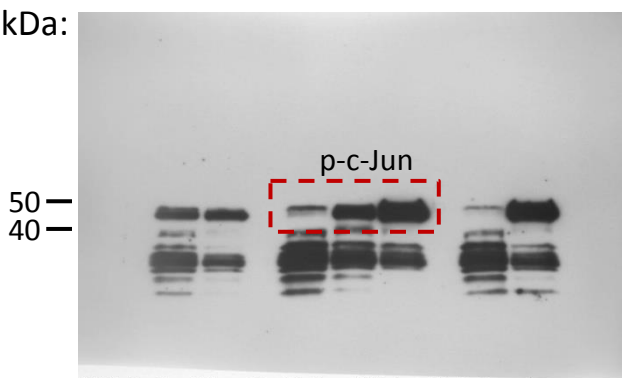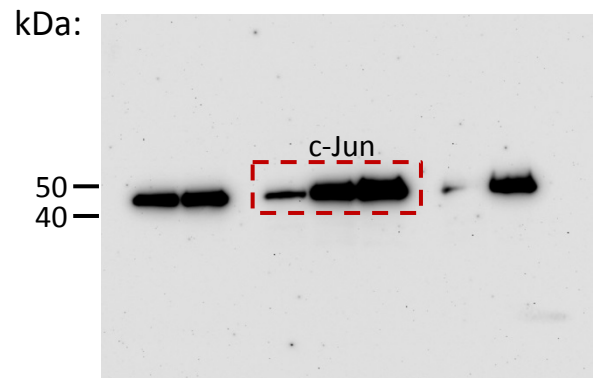

Source Data-Figure 3C

Supplement: Supplementary file 5 — Source Data for Figure 3 [file EMMM-10-e9003-s003.pdf]

PAX

DAPI

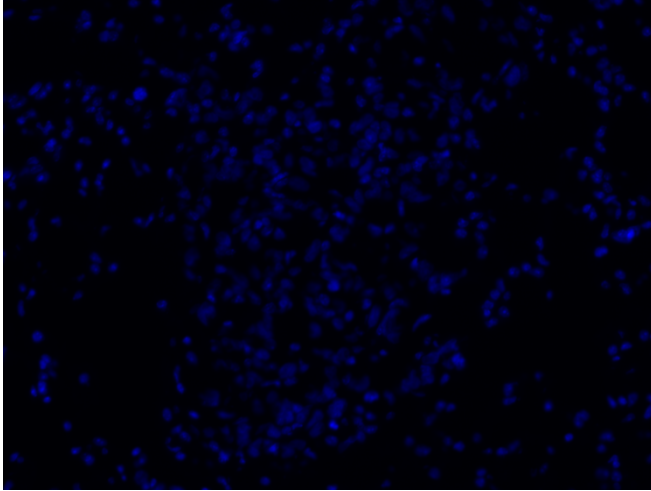

GFP

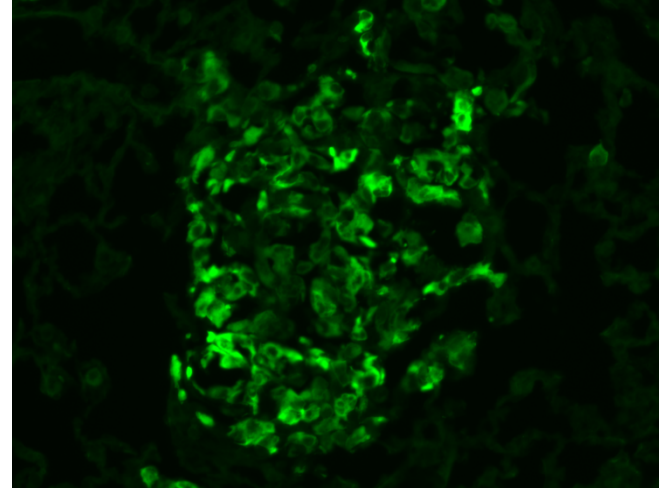

p-c-Jun

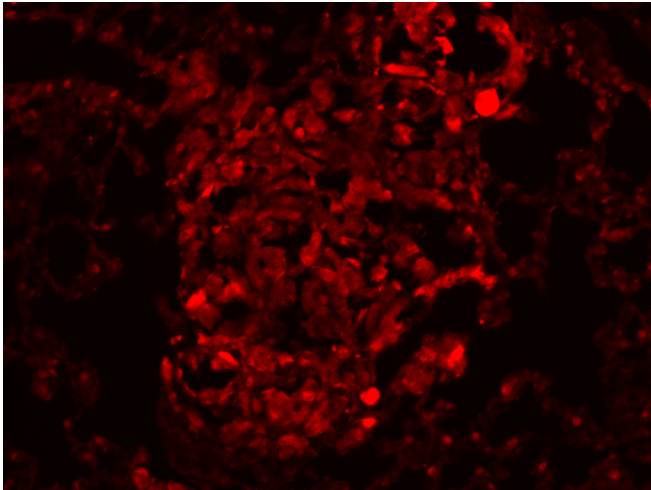

Merge

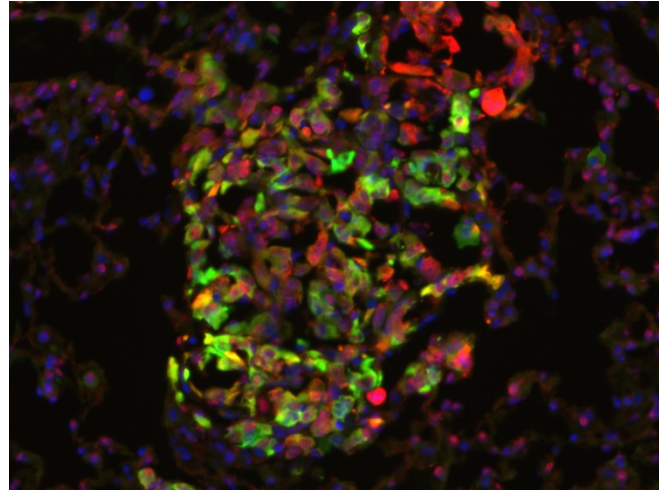

Vehicle

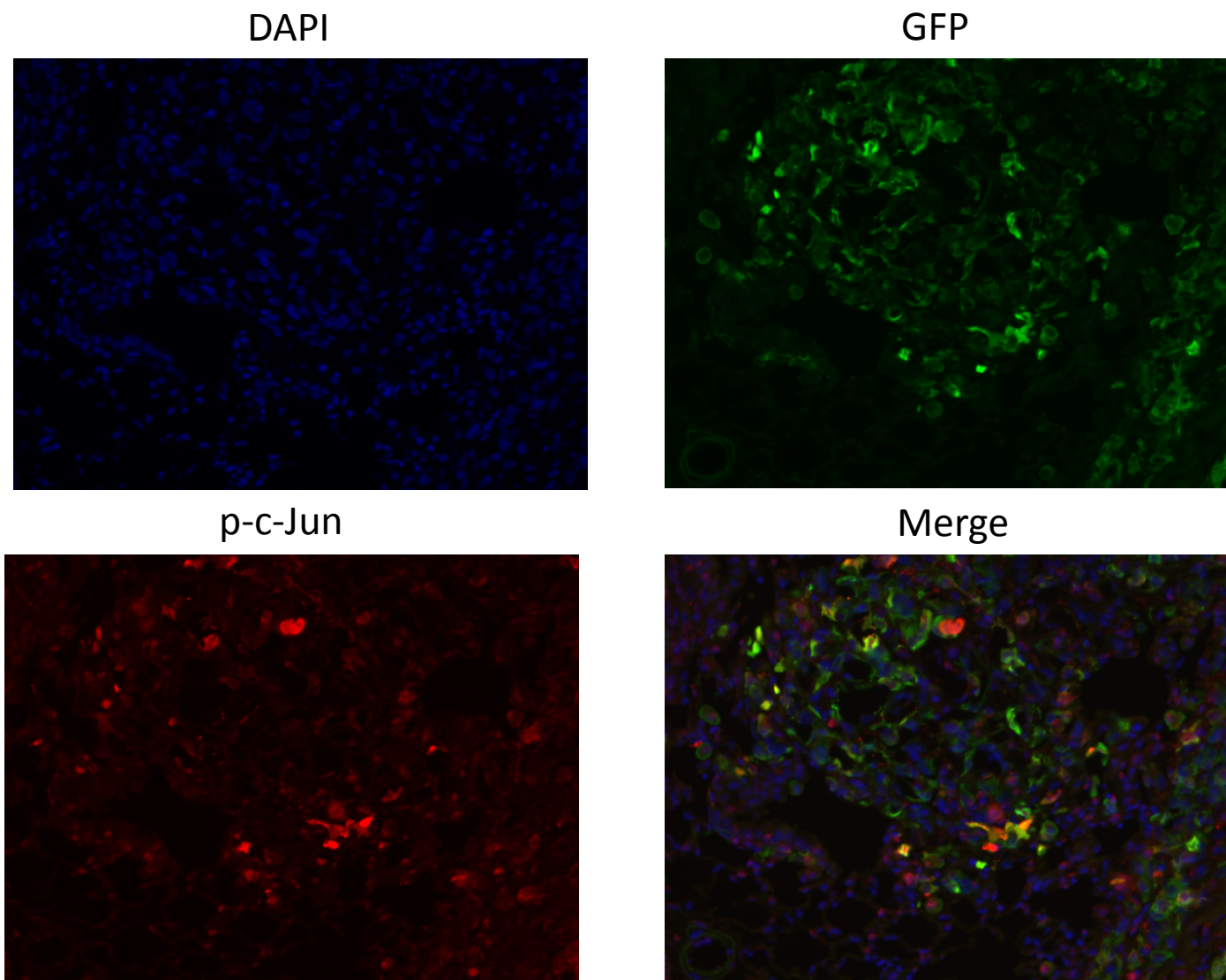

Source Data-Figure 5D

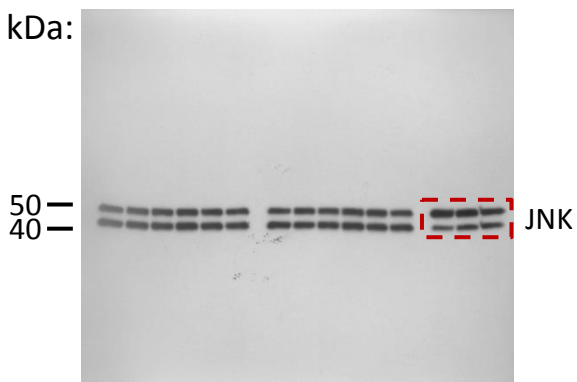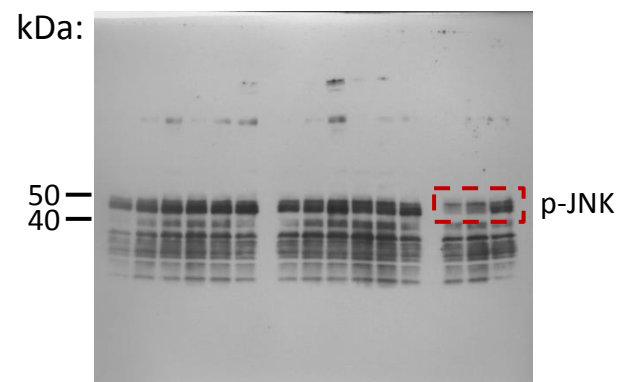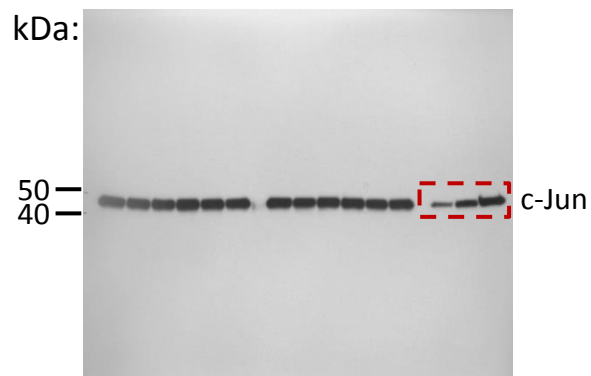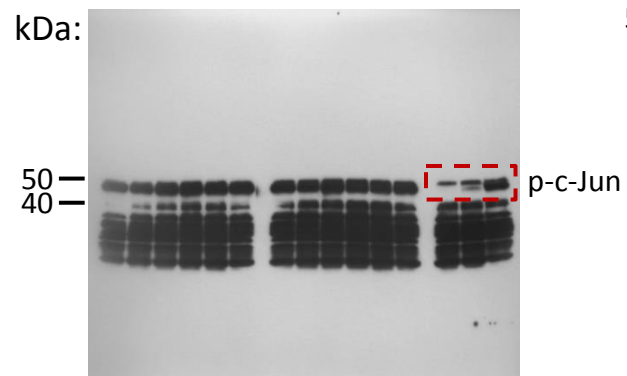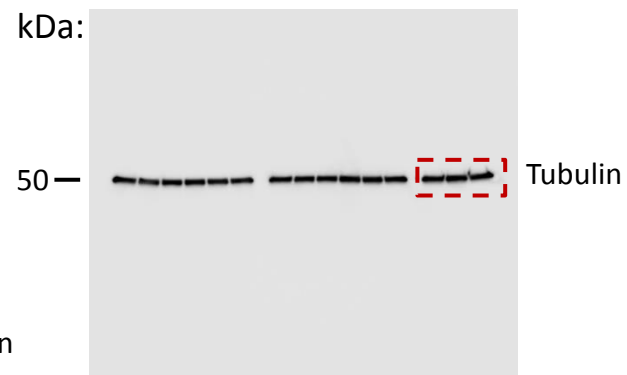

Source Data-Figure 5F

Supplement: Supplementary file 6 — Source Data for Figure 5 [file EMMM-10-e9003-s004.pdf]
